# Parental modelling and interpersonal support in relation to moderate physical activity and vigorous physical activity in 9–12-year-olds: A cross-sectional DEPASS study

**DOI:** 10.12688/openreseurope.21538.1

**Published:** 2025-10-28

**Authors:** Flávio Ferreira, Kieran Dowd, Alan Coffey, Greet Cardon, Evelien Iliano, Jan Dygrýn, Jana Pelclová, António Labisa Palmeira

**Affiliations:** 1 Lusófona University of Humanities and Technologies Faculty of Physical Education and Sport, Lisbon, Lisbon, Portugal; 2 Department of Sport and Health Sciences, Technological University of the Shannon Midlands Midwest Faculty of Science and Health, Athlone, County Westmeath, Ireland; 3 Department of Movement and Sports Sciences, Ghent University Faculty of Medicine and Health Sciences, Ghent, Flanders, Belgium; 4 Palacky University Olomouc Faculty of Physical Culture, Olomouc, Olomouc Region, Czech Republic; 5 Lusófona University of Humanities and Technologies Research Center on Sports Physical Education Exercise and Health, Lisbon, Lisbon, Portugal; 6 University of Porto Research Centre for Training Innovation and Intervention in Sport, Porto, Porto District, Portugal

**Keywords:** Accelerometry; Physical activity intensity; Interpersonal Support; Parental modelling

## Abstract

**Background:**

Moderate and vigorous physical activity (MPA and VPA) are both essential for child development and are influenced by both parental modelling and interpersonal support, yet most studies aggregate them into overall MVPA, potentially obscuring intensity-specific associations.

**Objective:**

This cross-sectional study examined intensity-specific associations between parental modelling, parental support, peer support, and teacher support and objectively measured child MPA and VPA.

**Methods:**

A total of 181 child–parent dyads from Belgium, Czechia, and Ireland were recruited between September 2023 and October 2024. Both children and their primary caregivers wore a hip-mounted ActiGraph wGT3X-BT accelerometer for eight consecutive days. Children and parents also completed validated Likert-scale questionnaires assessing interpersonal support and socioeconomic indicators. Associations between support variables and child PA outcomes were assessed using bivariate and partial correlations, controlling for confounders such as sex, country and socioeconomic status. Post-hoc analyses explored potential effects of extreme outliers (±2 SD from the mean).

**Results:**

Parental MPA was moderately correlated with child MPA (r≈0.30), whereas correlations for VPA were weaker (r≈0.20). Peer and teacher support showed no significant associations with MPA and VPA in primary analyses, although peer support showed a very weak correlation with child VPA after removing outliers.

**Conclusions:**

Parental modelling and co-participation were most strongly linked to children’s MPA, suggesting that family-based strategies may be more effective when targeting MPA. Peer support may have no role or a very limited one in VPA at this developmental stage, while teacher support showed no observable influence. These findings support the development of intensity-specific, multilevel interventions, but also highlight the need for longitudinal designs to clarify mechanisms and causality.

## Plain Language Summary

This study looked at how parents, friends, and teachers might influence how active children aged 9 to 12 are in their daily lives. Physical activity is very important for children’s growth and health. Doctors and health organizations recommend at least an hour of activity each day, yet most children around the world are not reaching this level. Being active helps children control their weight, strengthen their bones and muscles, and reduce the risk of health problems later in life.

Researchers in Belgium, Czechia, and Ireland asked 181 families to take part. Both children and their parents wore activity trackers for eight days to measure how much moderate activity (like walking or playing outside) and vigorous activity (like running or sports) they did. Children also filled in questionnaires about how much support children felt from parents, friends, and teachers.

The results showed that children were more active when their parents were active, especially for moderate activities. For example, if parents often walked or cycled, children were more likely to do the same. Vigorous activity, such as sports, showed weaker links to parents’ behaviour and was only slightly connected to friends’ encouragement. Teacher support was not found to make a difference in this study.

These findings suggest that the best way to help children be more active is through family involvement, especially when parents take part in activities with their children. Friends’ influence may become more important as children get older, but for this age group, parents are the strongest role models. This knowledge can help shape programs that aim to get children moving more often and more effectively.

## Introduction

Physical activity (PA) is essential for healthy growth and development in childhood. The World Health Organization 2020 guidelines recommend that children and adolescents aged five to 17 years accumulate at least 60 minutes per day of moderate to vigorous physical activity (MVPA), with muscle and bone strengthening at least three times per week ( Bull *et al.*, 2020). Despite this, over 80% of children and adolescents worldwide fall short of these targets, leaving many at increased risk for obesity, cardiometabolic disorders and suboptimal skeletal accrual during the critical years for lifelong bone health ( Guthold *et al.*, 2020). For this reason, identifying determinants of physical activity is crucial for informing both targeted interventions and broader policy strategies. Determinants may differ not only across demographic groups but also depending on the context in which adolescents live and grow. For instance, individual level factors such as self-efficacy, enjoyment of physical activity, and perceived competence often play a strong role in shaping behavior. Interpersonal influences including parental support, peer modeling, and encouragement can also significantly enhance or hinder participation. At the same time, institutional and environmental factors such as school curricula, the availability of safe recreational spaces, and access to sports facilities are important enablers or barriers. On a wider scale, community norms and policy level actions such as national guidelines, urban planning, and public health campaigns further influence the opportunities children have to engage in physical activity ( Kolovelonis *et al.*, 2024).

Among these interpersonal determinants, parental modelling has received particular attention as a powerful influence on children’s engagement in physical activity. Parental modelling refers to the extent to which parents demonstrate active lifestyles through their own behaviours, thereby serving as observable examples for their children ( Bringolf-Isler *et al.*, 2018). When children witness their parents engaging in regular PA, they may be more likely to view such behaviour as normative and attainable, which in turn can positively influence their own involvement in PA. This modelling effect operates through both direct imitation and the creation of an environment that supports active choices. Additionally, parental beliefs about children’s competence can interact with modelling behaviours to shape children’s motivation and perceived capability for PA ( Bois *et al.*, 2005).

Parental modelling, however, represents only one part of a broader social ecological framework. Within social ecological frameworks, interpersonal support from parents, peers and teachers is proposed as a principal influence on children’s activity alongside intrapersonal, environmental and policy factors ( Sallis *et al.*, 2000). Parental support has demonstrated robust links with child MVPA, yet most evidence has been derived from questionnaire-based PA measures that may overestimate associations ( Yao & Rhodes, 2015). When both parent and child activity are assessed by accelerometry, correlations fall to approximately 0.2 for overall parental support ( Sarker *et al.*, 2015; Trost & Loprinzi, 2011). Peer support has also demonstrated associations with PA levels in both pre-adolescents (commonly defined as ages 9–12) and adolescents (ages 10–19) ( Khan *et al.*, 2020), while teacher support has been shown to boost schooltime PA through quality physical education and classroom activity breaks, yet its unique contribution to total daily activity as captured by accelerometry is small and sometimes non-significant ( Sallis *et al.*, 2002; Sheridan *et al.*, 2014).

The intensity of PA shapes distinct health outcomes. Moderate-intensity PA (MPA, 3.0–5.9 metabolic equivalents [METs]) underpins weight management, glycemic control, and lipid metabolism, whereas vigorous-intensity PA (VPA, ≥ 6.0 METs) confers additional benefits for aerobic capacity and musculoskeletal health ( Herrmann *et al.*, 2024; Poon *et al.*, 2021; WHO, 2020). Regular MPA is consistently linked to reductions in adiposity, improved glycemic regulation, and favorable changes in lipid profiles, while VPA and interval-based modalities elicit superior gains in cardiorespiratory fitness and glucose control ( Gallardo-Gómez *et al.*, 2024; Mendes *et al.*, 2019; Syeda *et al.*, 2023). High-impact activities such as running and jumping stimulate osteogenesis, supporting accrual of peak bone mass, the strongest predictor of fracture risk in later life ( Chevalley & Rizzoli, 2022; Miao *et al.*, 2025; Ng *et al.*, 2023). Accurate assessment of intensity and duration is therefore vital. Waist-worn accelerometry provides objective, epoch-by-epoch measurement that can be classified into light, moderate and vigorous intensity bands using validated count thresholds ( Evenson *et al.*, 2008). By contrast, self-report questionnaires, though useful for capturing context and perceptions, are prone to recall error and social desirability bias and yield only modest correspondence with accelerometer-derived activity ( Duncan *et al.*, 2005). Moreover, questionnaire-based studies often report stronger associations between interpersonal support and activity than those observed when both support and PA are measured using devices.

Most research to date aggregates MVPA into a single composite as seen in the meta-analysis by Petersen *et al.* (2020), masking potential differences in how support relates to each intensity. A handful of studies hint that parental modelling may be more closely tied to MPA behaviors such as walking and non-competitive play (e.g., playground games, casual bike rides, or shooting hoops, while peer invitations and structured opportunities may be more critical for VPA participation ( Mattocks *et al.*, 2007). However, intensity-specific pathways remain underexplored, especially in studies combining objective measures of activity with questionnaire-based assessments of support.

Children aged 9-12 occupy a developmental niche in which autonomy is increasing yet caregiver influence remains strong ( Yao & Rhodes, 2015). In this age group, parental modelling of active behavior is likely the dominant support factor, whereas teacher encouragement is confined to the school day and may play a supplementary role. Peer support begins to emerge as a motivator for VPA such as team sports but exerts less influence over overall MVPA than parental modelling ( Li-juan *et al.*, 2017).

The present study draws on a subset of data from the European COST Action DE-PASS (Determinants of Physical Activities in Settings), which aims to identify, understand, and measure the factors that promote, sustain, or inhibit PA behaviors across the lifespan and in various settings. One of its dedicated working groups ( Palmeira *et al.*, 2024) focused specifically on children aged 9–12 and developed a harmonized set of procedures and protocols to standardize collection of PA determinant data across participating countries, ensuring methodological consistency and cross-national comparability. Currently, DE-PASS data are being processed from ten countries, forming a comprehensive pan-European database of PA determinants and behaviors. Within this framework, determinants are examined at multiple levels, including individual (psychological and behavioral), interpersonal, institutional, and policy/societal levels, thereby capturing a broad spectrum of influences on physical activity participation. This study focuses on a research question not addressed in the main DE-PASS analyses, thereby offering novel insights within the project's broader framework.

This dataset was collected by deploying accelerometers to quantify children’s and parental daily MPA and VPA to measure parental modelling, and validated questionnaires to assess parent support, peer support and teacher support ( Palmeira *et al.*, 2024). Correlations among objectively measured MPA and VPA, parental modelling and each support type, were examined both separately and in aggregate. The aim of this study is (i) to verify if parental support will show the strongest associations with children’s MPA, (ii) peer support is positively correlated with MPA and VPA, (iii) teacher support will have little association with MPA and VPA and (iv) parental modelling is positively correlated with child MPA and VPA, with a stronger relationship for MPA than for VPA.

## Methods

### Study design and setting

This cross-sectional study was conducted in primary schools across three European countries (Belgium, Czechia, and Ireland) between September 2023 and October 2024. Ethical approval for this study was obtained independently in each participating country. In Portugal, approval was granted by the CEFADE Ethics Committee, Faculty of Physical Education and Sport, Lusófona University (Ref. CEFADE 34/2023). In Ireland, approval was obtained from the Research Ethics Committee of the Technological University of the Shannon (Ref. 20221201). In Belgium, approval was granted by the Ethics Committee of Ghent University (Ref. 2023-106). In Czechia, approval was provided by the Ethics Committee of the Faculty of Physical Culture, Palacký University Olomouc (Ref. No. 90/2023). All procedures were conducted in accordance with the Declaration of Helsinki, and written informed consent was obtained from parents/guardians and assent from children prior to participation. All procedures followed the DE-PASS Proof-of-Concept Standard Operating Procedures (SOP), and the reporting adhered to the Strengthening the Reporting of Observational Studies in Epidemiology (STROBE) guidelines ( von Elm *et al.*, 2007) (In supplementary 2).

### Participants

Children aged 9–12 years without diagnosed mental or physical conditions likely to alter habitual PA were eligible, provided their primary caregiver (the parent or guardian most familiar with their daily routines) also consented to participate. Recruitment was school-based (both public and private) in collaboration with school principals, initiated by a researcher-led presentation to the parent community; interested families returned signed consent forms prior to data collection.

### Data collection procedures

From each family, one study child, one primary caregiver, and one sibling (aged 8–17 years) were invited to participate, provided written informed consent (caregiver and siblings aged ≥18 years) and assent (children and younger siblings) were obtained. Families were allocated anonymized ID codes to ensure confidentiality and facilitate data linkage across measures. All procedures were approved by local ethics committees prior to implementation.

At the scheduled data collection session, families attended a face-to-face meeting with trained researchers, usually hosted in the school environment. During this visit, general family information was collected, and children’s height and weight were measured following standardized anthropometric protocols. Both the study child and primary caregiver completed validated determinant questionnaires, with assistance provided if required. To capture device-based physical activity behaviors, accelerometers were distributed to the study child, one sibling, and the primary caregiver, along with an accelerometer diary to record wear times, removals, and structured physical activity. Participants were instructed to wear the devices for eight consecutive days. Families received a study care pack containing all instructions, consent forms, and return materials. Reminder text messages were sent to caregivers on days three, four, and eight to support compliance and timely return of devices and documentation. Returned data was checked for completeness before entry into the centralized DE-PASS database.

### Accelerometer protocol

Participants wore an ActiGraph wGT3X-BT monitor (ActiGraph Corp., Pensacola, FL) affixed to a latex-free elastic belt at the right hip for eight days, removing it only for water-based activities or sleep. The ActiGraph devices were initialized using the ActiLife 6.13.4 software to sample at a 30 Hz rate. All family members received an illustrated diary and optional SMS reminder on days three and four to reinforce wear compliance. Devices were initialized with a delayed start (03:00 on the second day) and programmed for automatic stop after eight days, with sleep mode enabled and LED display suppressed to minimize distraction. Unique DE-PASS ID codes were entered during initialization to ensure correct data linkage.

Upon return, raw .agd and .gt3x files were downloaded following the DE-PASS folder structure and naming convention. Data were processed with a 10-s epoch, three-axis acceleration, step count, light sensor and inclinometer channels. For children, non-wear was defined by the Troiano algorithm ( Troiano *et al.*, 2008) as ≥ 60 minutes consecutive zero counts with allowance for up to 2 minutes nonzero interruptions. Valid days required ≥ 480 minutes of wear time, with ≥ 3 valid weekdays and ≥ 1 valid weekend day required to meet the inclusion criteria. For parents, non-wear time was defined as ≥ 60 minutes consecutive zero counts. Files were examined for MPA and VPA in ActiLife using the Evenson *et al.* (2008) cut-points for children and the Troiano *et al.* (2008) cut-points for parents. Average daily MPA and VPA were calculated across valid days. While many studies process data in 60-s epochs, this approach can under-detect brief and intermittent bouts of activity, particularly vigorous bursts. By processing at a 10-s epoch, our protocol aimed to better preserve these patterns, although this may limit direct comparability with studies using longer epochs. This protocol was adapted from Decelis *et al.* (2014). All downloaded data were securely stored in GDPR-compliant institutional servers following DE-PASS protocols, with dual verification and password-protected master ID linkage files.

### Support assessment protocol

Both parents and children completed questionnaires. The child survey was an adapted version of the Parental Encouragement and Support for Children’s PA scale (PEACH; Ommundsen *et al.*, 2008). The parent survey comprised socio-demographic items from the Health Behaviour in School-aged Children questionnaire (HBSC; Roberts *et al.*, 2009) filled by the parent. Both questionnaires followed the adaptation procedure described by Palmeira *et al.* (2024) and were delivered either on paper. Children PEACH questionnaire yielded three subscales: parental support, peer support and teacher support. Responses were recorded on a four-point Likert scale (1 = Hardly ever or never to 4 = Every day). The original version of the instrument demonstrated good internal consistency (Cronbach’s α = .72–.89) and acceptable test–retest reliability (ICC = .65–.80) as reported in ( Palmeira *et al.*, 2024).

Missing or incomplete questionnaires were first addressed on site by the researcher’s assistance; any forms still unfinished or unreturned prompted a follow-up phone call. Instruments that remained incomplete after these recovery steps were excluded from analysis in accordance with the standard operating procedures.

### Statistical methods

Participants with no valid child and parent accelerometry data or child questionnaire for the same entry, were removed from further analysis. Questionnaire responses for parental, peer, and teacher support were coded on an ordinal scale (1 = “Hardly ever or never” to 4 = “Every day”), and subscale scores were computed as the mean of their respective items (five for parental support, three each for peer and teacher support). MPA and VPA data were transformed (square root) to normalize data and Shapiro–Wilk tests were used to assess the normality of each variable’s distribution, and Levene’s test was used to assess equality of variances, as recommended by Field (2013). The distribution of VPA remained non-normal even after a square root transformation. Average daily MPA and VPA (minutes per day) were calculated by dividing the total accumulated minutes by the number of valid observation days.

To identify potential socioeconomic confounders, bidirectional associations between each support variable, PA outcome (MPA and VPA minutes/day, % of total) and a set of candidate covariates were examined. Candidates were screened via point-biserial correlation using Tabachnick & Fidell, 2007 recommendations (full results in supplementary 1, socioeconomic questions in Table 1); only variables showing p < .05 associations with both an independent (support or Parent MPA and VPA) and a dependent (child MPA and VPA) variable were retained for adjustment. Child sex, country and age were directly included as covariates, given their established role as potential confounders ( Carson *et al.*, 2020; Rodríguez-Rodríguez *et al.*, 2020). Regression models were then estimated with and without these covariates to assess their impact on effect estimates.

**Table 1.** Demographic, Socioeconomic, and Accelerometry-Derived Physical Activity Characteristics of child–parent Dyads.

Characteristic	Child (N = 172)	Parent (N = 172)
**Demographics**		
**Age, mean (SD), years**	10.9 (1.6)	42.3 (5.8)
**Country of origin, n (%)**		
**Belgium**	59 (34.3)	
**Czechia**	67 (39.0)	
**Ireland**	46 (26.5)	
**Sex, n (%) ^#^**		
**Male**	86 (50.0)	40 (23.3)
**Female**	83 (48.3)	129 (75.0)
**Education ≥ Bachelor’s, n (%)**	—	123 (71.5)
**Socioeconomic**		
**Cars owned, n (%)**		
**0**	—	3 (1.7)
**1**	—	44 (25.6)
**2**	—	110 (64.0)
**≥ 3**	—	8 (4.7)
**Own room, n (%)**		
**Yes**	133 (77.3)	—
**No**	34 (19.8)	—
**Travels abroad, n (%)**		—
**None**	18 (10.5)	
**Once**	75 (43.6)	
**Twice**	46 (26.7)	
**> Twice**	28 (16.3)	
**Computers, n (%)**		
**0**	—	4 (2.3)
**1**	—	32 (18.6)
**2**	—	41 (23.8)
**> 2**	—	90 (52.3)
**Dishwasher, n (%)**	—	No 14 (8.1) Yes 153 (89.0)
**Bathrooms, n (%)**		
**1**	80 (46.5)	
**2**	55 (32.0)	
**> 2**	32 (18.6)	
**Physical Activity, mean (SD)**		
**MPA, minutes/day**	33.3 (10.5) *	42.87 (16.4) *
**MPA (% total PA)**	4.0 (1.3)	4.9 (1.9) *
**VPA, minutes/day**	18.8 (10.8)	4.5 (8.3)
**VPA (% total PA)**	2.2 (1.2)	0.5 (0.9)
**Social Support**		
**Peer support, mean (SD)**	2.8 (0.8)	—
**Parental support items, mean (SD)**		—
**Take you to exercise**	2.8 (1.0)	—
**Watch you take part in exercise**	3.0 (1.0)	—
**Exercise or play sports with you**	3.1 (0.9)	—
**Tell you to exercise**	2.8 (1.0)	—
**Tell you that exercise is good for your health**	2.8 (1.1)	—
**Teacher support items, mean (SD)**		—
**Talk about exercise in lessons**	3.0 (1.0)	—
**Organize or play games apart from PE**	3.2 (0.9)	—
**Tell you to exercise or play sports**	2.5 (0.9)	—

*Statistically significant difference between Male and Female. **Note:** Totals aren’t 100% since some entries were blank. # Three participants did not answer

Descriptive statistics (means, standard deviations, and ranges for continuous variables; frequencies and percentages for categorical variables) characterized the sample and key measures. Correlations involving Support as the independent variable were calculated using Spearman’s rank-order coefficient, and those with Parent MPA and VPA as the independent variable used Pearson’s product-moment coefficient; for any variables that violated normality or homoscedasticity assumptions required by Pearson’s method, Spearman’s coefficient was applied instead.

Internal consistency of each support subscale in this study was assessed using McDonald’s omega (ω) ( McDonald, 1999), which revealed variable internal consistency. McDonald’s omega was acceptable for peer support (ω = .70) but lower for parental support (ω = .51) and teacher support (ω = .38). Since teacher and Parental support dimensions cannot be used as a single determinant, individual questions were used for the correlation analysis.

Post-hoc analyses were conducted by removing outliers from the parent–child MPA and VPA dataset, defined as values falling outside the mean ± 2 standard deviations. This approach aimed to normalize the distribution and explore whether excluding extreme cases (e.g. parents with exceptionally high VPA) would change the association patterns. All relevant variables were reanalyzed using the square root transformation procedures previously described.

Data on MVPA was not included in the present analyses, as this study was designed to focus specifically on distinguishing between MPA and VPA. Analyses of MVPA will be conducted in future work.

All analyses were conducted using JASP version 0.19.2 with a two-tailed α = .05.

## Results

### Descriptive statistics

**Figure 1.**
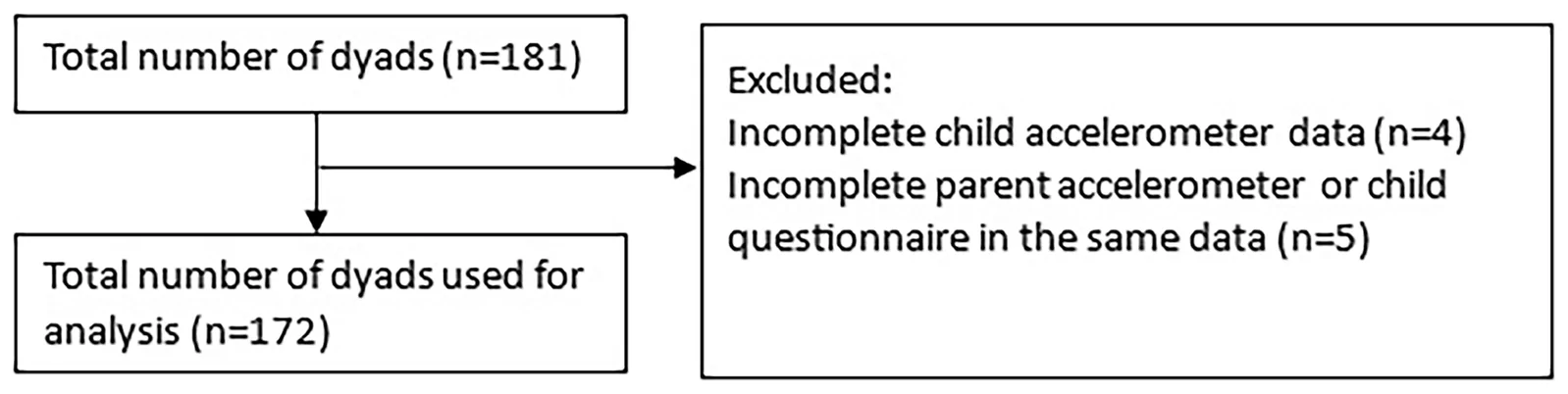
Dyads flow chart.

There were originally 181 dyads, resulting in a final sample size of 172 after removing dyads that did not meet the eligibility criteria ( Figure 1). Sample characteristics and accelerometry outcomes for the 172 child–parent dyads included in the final analysis are presented in Table 1. A post-hoc analysis was conducted using G*Power version 3.1.9.7 for a Two-Tailed Point biserial model reaching 80% for |ρ|=0.19. Children and parents were of very similar age groups, sample country distribution was balanced, child sex was very balanced, while parental sex was much more female dominant. The overall group is highly educated and of a high socioeconomic status. In terms of normality, Parent VPA in minutes and % stood out as having a non-normal distribution even after transformation which remained right skewed. In terms of sex differences, they were only found in accelerometry in both child and parents and only in MPA.

### Correlation analysis

Pearson correlation analyses between parent and child PA ( Table 2) demonstrated small to moderate positive associations across corresponding intensity levels (e.g., Parent MPA minutes were associated with children MPA, but Parent VPA was not associated with children MPA). When controlling for child sex, the correlation between child MPA minutes and parental VPA minutes became significant (ρ = .156, 95% CI [0.004, 0.295], p = .043).

**Table 2.** Pearson correlation matrix depicting associations between parent and child MPA and VPA metrics.

	Parent MPA (minutes)	Parent MPA (%)	Parent VPA (minutes) ^a^	Parent VPA (%) ^a^
**Child MPA (minutes)**	**.298** [.154, .429] < .001	**.253** [.107, .389] < .001	.146 [–.005, .290] .058	.141 [–.010, .285] .068
**Child MPA (%)**	**.291** [.147, .423] < .001	**.342** [.202, .468] < .001	.032 [–.119, .181] .682	.045 [–.106, .194] .559
**Child VPA (minutes)**	.092 [–.059, .239] .233	.030 [–.121, .180] .698	**.240** [.093, .377] .002	**.230** [.082, .367] .003
**Child VPA (%)**	.091 [–.060, .238] .239	.081 [–.071, .228] .295	**.198** [.049, .338] .010	**.197** [.048, .338] .010

a- Spearman correlation. **Note:** Results are presented as correlation coefficients with corresponding 95% confidence intervals and p-values.

Adjustment for the tested confounders did not alter the statistical significance of the associations. The full correlation matrix with partial correlations can be found in supplementary 1. Associations between child-reported social support and PA ( Table 3) were generally nonexistent to weak. After adjusting for child sex, some additional significant associations emerged. The correlation between the item ‘Exercise or play sports with you’ and child MPA % increased (ρ = 0.188, 95% CI [0.041, 0.337], p = 0.014). Furthermore, new significant associations with ‘Exercise or play sports with you’ were observed for child MPA minutes (ρ = 0.167, 95% CI [0.022, 0.301], p = 0.030), and child VPA % (ρ = 0.157, 95% CI [0.004, 0.299], p = 0.041).

**Table 3.** Spearman correlations between child-reported social support (parental, peer, teacher) and children’s MPA and VPA outcomes.

	Peer Support	(Parent) Take you to exercise	(Parent) Watch you take part in exercise	(Parent) Exercise or play sports with you	(Parent) Tell you to exercise	(Parent) Tell you that exercise is good for your health	(Teacher) Talk about exercise in lessons	(Teacher) Organize or play games apart from PE	(Teacher) Tell you to exercise or play sports
**Child MPA (minutes)**	.035 [–.127, .194] .649	.104 [–.059, .255] .173	.007 [–.166, .145] .929	.122 [–.014, .267] .110	.012 [–.135, .162] .872	.061 [–.077, .207] .424	–.068 [–.221, .088] .375	–.137 [–.273, .004] .074	–.025 [–.178, .124] .745
**Child MPA (%)**	.039 [–.117, .184] .614	.017 [–.145, .168] .824	–.109 [–.266, .050] .154	**.159** [.022, .309], .037	–.031 [–.176, .121] .686	–.013 [–.151, .131] .867	–.098 [–.246, .057] .201	–.037 [–.182, .113] .632	–.025 [–.166, .112] .748
**Child VPA (minutes)**	.011 [–.147, .163] .889	.024 [–.134, .180] .757	–.009 [–.160, .148] .907	.117 [–.033, .263] .125	–.022 [–.176, .118] .775	.011 [–.131, .167] .883	.020 [–.141, .168] .799	–.096 [–.240, .052] .210	.014 [–.148, .168] .857
**Child VPA (%)**	-.003 [–.162, .141] .969	-.035 [–.187, .124] .651	–.058 [–.207, .100] .448	.140 [–.014, .288] .067	–.034 [–.184, .112] .662	–.035 [–.170, .114] .650	.011 [–.151, .163] .886	–.044 [–.192, .111] .568	.018 [–.135, .171] .816

**Note:** Results are presented as correlation coefficients with corresponding 95% confidence intervals and p-values.

When controlling for socioeconomic determinants, specifically questions “Cars owned” and “Bathrooms”, the results were similar, with some already marginally significant values losing significance. The complete correlation matrix with partial correlations is available in supplementary 1.

Removing outlier cases did not materially change the parent–child PA correlations. However, after outlier removal, peer support showed a new, weak significant correlation with child VPA minutes (ρ = .171, 95% CI [0.006, 0.327], p = .049).

## Discussion

This study revealed intensity-specific associations between interpersonal influences and children's PA, with particularly strong links between parental behaviour and MPA in children. Consistent with the fourth aim and previous research, parental MPA was most strongly correlated with child MPA (r = .342, p < .001), while parental VPA showed only a weak correlation with child VPA (ρ = .197, p = .010), reinforcing the role of parental modelling primarily in moderate activity. These findings are in line with Sigmundová *et al.* (2024), who also reported stronger parent–child associations for MPA (ρ = .35) compared to VPA. MPA, such as walking or casual play, are more accessible for co-participation or observation ( Gustafson & Rhodes, 2006), and adults are more likely to engage in them themselves ( Trost *et al.*, 2003; Yao & Rhodes, 2015). These patterns may help explain why parental modelling exerts a stronger influence at the moderate intensity level. Showing that MPA behaves differently from VPA which has a considerably greater variability, suggesting that distinct strategies may be required to effectively enhance VPA or MPA.

In support of aim one which predicted that parental support would show the strongest associations with children’s MPA, perceived parental support through the item "Exercise or play sports with child", was positively associated with child MPA % (r = .159, p = .037). This item reflects co-participation, which has been identified by Pyper *et al.* (2016) as a key predictor of child physical activity. Fitness levels typically decline with age, and this reduction in capacity can make participation in vigorous activities more difficult to sustain ( Sun *et al.*, 2013). Safety considerations, such as increased risk of injury or discomfort associated with high exertion, may also discourage parents from engaging in VPA with their children or alone. In contrast, MPA such as walking, cycling at a casual pace, or active play are easier to integrate into daily routines, better align with the fitness levels of most parents, and pose fewer barriers. MPA is therefore more practical for parents to adopt and to model within family contexts, making it a more promising focus for family-based interventions. However, Trost *et al.* (2003) suggested caution, since parental support alone may not be sufficient to overcome environmental or motivational barriers, and broader multilevel strategies may be required. These findings on parent support and modelling highlight the importance of distinguishing between types and intensities of activity.

Our second aim, that peer support would be associated with child VPA, was not supported by the primary analysis, which revealed no significant correlation. This result may reflect the developmental stage of the children in this sample, aged 9 to 12 years, who are still primarily influenced by parental guidance and support, as described by Beets *et al.* (2010). While peer support is theorized to become more influential with age, it may not yet play a prominent role in vigorous activity at this stage of development. A post hoc exploratory analysis, conducted after the removal of outliers, which consisted mainly of dyads with exceptionally high VPA, did reveal a weak but statistically significant correlation between peer support and child VPA minutes (ρ = .171, p = .049). Although this suggests a possible emerging influence of peers under certain conditions, it should be interpreted cautiously. Peer influence on VPA may operate indirectly through mechanisms such as shared enjoyment or self-efficacy rather than direct encouragement ( Chen *et al.*, 2017), and as mentioned before it may only gain relevance closer to adolescence ( Davison & Jago, 2009; Zhou *et al.*, 2023). The post hoc findings may reflect distinct behavioural patterns among children with lower parental VPA exposure, but they do not alter that, in this age group, peer support by itself was not a significant predictor of VPA. This indicates that the timing and tailoring of interventions should be based on developmental stage, and that for this age group peer focused interventions may not be the best suited.

Lastly, the third aim on teacher support was confirmed since no significant association with physical activity was found across any intensity level. This contrasts with some intervention studies, such as Eather *et al.* (2013), which demonstrated increased physical activity following teacher-supported interventions. However, those studies differ in both context and methodology. The study by Eather and colleagues focused on a structured intervention in an Australian setting, whereas this study evaluated naturally occurring behaviours in a European sample. Teacher support may vary depending on national education systems, and as suggested by Lin *et al.* (2024), because of this, it may be inconsistently linked to physical activity outcomes across studies. In contexts where teachers are not expected or resourced to promote activity outside of physical education, their influence may be negligible.

Overall, these findings suggest that interpersonal influences on child physical activity vary by intensity and, in the case of teacher support, by context. Parental modelling is especially relevant for MPA, while peer support probably has no major influence on this age group. The role of teachers appears non-relevant in this sample, potentially due to systemic or cultural limitations within the education system.

## Limitations

This study has several limitations. The cross-sectional design prevents any inference of causality, making it impossible to determine the directionality of observed associations. The sample was primarily composed of families from higher socioeconomic backgrounds, European countries and mostly mothers (75%), which may limit the generalizability of the findings to other sex, cultural or socioeconomic groups. Other environmental factors such as physical environment or school policies which may potentially influence PA were also not accounted for in this study. The sample is from a small set of schools sometimes even the same classes, therefore having a potential cluster (especially for teacher support) effect violating the assumption of them being independent observations, but since that data is not shared within data collection agents for confidentiality reasons, that analysis was not possible.

Although the original parental, peer, and teacher support instruments had acceptable reliability in other research, the internal consistency of the parental and teacher support subscales was suboptimal in this study. As a result, only individual items could be used, which may have limited the ability to capture the full scope of interpersonal support constructs. It is possible that the age group studied, the method of questionnaire deployment or translation method was not optimal for capturing these two support constructs, and children’s tendency to provide socially desirable responses may have introduced bias. Future research should refine and validate more comprehensive measurement tools for interpersonal influences on child activity.

The sample size, while adequate for detecting moderate associations, lacked power to identify very weak correlations. Additionally, the exploratory nature of some secondary analyses, such as stratifying by activity level or socioeconomic markers, means those findings should be interpreted with caution. The study's age range also limits its applicability to other developmental stages, particularly adolescents, where different social dynamics may be more relevant.

## Conclusions

This study highlights the importance of considering the type of physical activity intensity when examining interpersonal influences on child activity. Parental modelling appears to have the most substantial influence on MPA, supporting the value of interventions that promote habitual family-based movement. Peer support did not seem to contribute to vigorous activity or at best play a limited role for this age group. Teacher support was not significantly associated with physical activity in this sample, possibly due to contextual factors within European education systems.

Future research should use longitudinal designs and refined, validated instruments to better understand the direction and mechanisms of these relationships, with particular attention to clearly distinguishing between VPA and MPA in order to determine which strategies are most effective for each intensity. Interventions should consider tailoring strategies to the specific intensity of physical activity and the developmental stage of the child, recognizing that different social influences may be relevant at different times and for different types of activity.

## Extended data

Open Science Framework. DE-PASS MPA and VPA cross-sectional supplementary data. OSF, https://osf.io/qc8g2/?view_only=2ec73bad89674857859d142f4e6f7d7c ( Ferreira *et al.*, 2025)

This project contains the following extended data:

•    Supplementary 1 (Correlation matrices: partial correlations with socioeconomic covariates).

•    Supplementary 2 (STROBE checklist).

Data are available under the terms of the Creative Commons Attribution 4.0 International license (CC BY 4.0).
