# Moderate and vigorous physical activity show distinct familial and interpersonal association patterns in 9–12-year-olds: A cross-sectional DE-PASS study

**DOI:** 10.12688/openreseurope.21538.2

**Published:** 2026-03-10

**Authors:** Flávio Ferreira, Kieran Dowd, Alan Coffey, Greet Cardon, Evelien Iliano, Jan Dygrýn, Jana Pelclová, António Labisa Palmeira, Eco J. C. de Geus

**Affiliations:** 1 Lusófona University of Humanities and Technologies Faculty of Physical Education and Sport, Lisbon, Lisbon, Portugal; 2 Department of Sport and Health Sciences, Technological University of the Shannon Midlands Midwest Faculty of Science and Health, Athlone, County Westmeath, Ireland; 3 Department of Movement and Sports Sciences, Ghent University Faculty of Medicine and Health Sciences, Ghent, Flanders, Belgium; 4 Palacky University Olomouc Faculty of Physical Culture, Olomouc, Olomouc Region, Czech Republic; 5 Lusófona University of Humanities and Technologies Research Center on Sports Physical Education Exercise and Health, Lisbon, Lisbon, Portugal; 6 University of Porto Research Centre for Training Innovation and Intervention in Sport, Porto, Porto District, Portugal; 7 Department of Biological Psychology, Faculty of Behavioural and Movement Sciences, Vrije Universiteit Amsterdam, Amsterdam, The Netherlands

**Keywords:** Accelerometry; Physical activity intensity; Interpersonal Support; Parental modelling

## Abstract

**Background:**

Research on children’s physical activity often examines interpersonal associations but commonly aggregates MPA and VPA into a single MVPA outcome. This approach may obscure intensity-specific association patterns, particularly in intergenerational analyses.

**Methods:**

This cross-sectional study used harmonized data from the European DE-PASS project, including children aged 9–12 years and one of their parents. Waist-worn accelerometry was used to assess MPA and VPA. Perceived parental, peer, and teacher support were measured using validated questionnaires. Intensity-specific associations between parent and child physical activity and between interpersonal support variables and children’s activity were examined, including sex-stratified and socioeconomic sensitivity analyses.

**Results:**

Parent–child associations differed by activity intensity. Parent–child resemblance was stronger for MPA than for VPA, while associations involving VPA were weaker and less consistent. Parental co-participation support was positively associated with children’s MPA, whereas peer and teacher support showed limited or no associations with children’s activity at either intensity. Sex-stratified analyses indicated similar MPA associations across boys and girls, while associations for VPA were not observed among girls. Aggregating MPA and VPA into MVPA would have obscured these intensity-specific patterns.

**Conclusions:**

These findings show that interpersonal associations with children’s physical activity differ by intensity and support treating MPA and VPA as distinct outcomes in intergenerational analyses. Family-based approaches focusing on MPA may be particularly relevant during late childhood, while different strategies may be needed to address VPA.

## Plain Language Summary

This study looked at how parents, friends, and teachers might influence how active children aged 9 to 12 are in their daily lives. Physical activity is very important for children’s growth and health. Doctors and health organizations recommend at least an hour of activity each day, yet most children around the world are not reaching this level. Being active helps children control their weight, strengthen their bones and muscles, and reduce the risk of health problems later in life.

Researchers in Belgium, Czechia, and Ireland asked 181 families to take part. Both children and their parents wore activity trackers for eight days to measure how much moderate activity (like walking or playing outside) and vigorous activity (like running or sports) they did. Children also filled in questionnaires about how much support children felt from parents, friends, and teachers.

The results showed that children were more active when their parents were active, especially for moderate activities. For example, if parents often walked or cycled, children were more likely to do the same. Vigorous activity, such as sports, showed weaker links to parents’ behaviour and was only slightly connected to friends’ encouragement. Teacher support was not found to make a difference in this study.

These findings suggest that the best way to help children be more active is through family involvement, especially when parents take part in activities with their children. Friends’ influence may become more important as children get older, but for this age group, parents are the strongest role models. This knowledge can help shape programs that aim to get children moving more often and more effectively.

## Introduction

Physical activity is essential for healthy growth and development in childhood. The World Health Organization 2020 guidelines recommend that children and adolescents aged 5 to 17 years accumulate at least 60 minutes per day of moderate to vigorous physical activity (MVPA), with muscle and bone strengthening at least three times per week ( Bull *et al.*, 2020). Despite this, over 80% of children and adolescents worldwide fall short of these targets, increasing risk for obesity, cardiometabolic disorders, and suboptimal skeletal accrual during key developmental years ( Guthold *et al.*, 2020). Identifying associations with physical activity remains important for informing intervention development and policy, recognizing that influences operate across individual, interpersonal, school, and environmental levels ( Kolovelonis *et al.*, 2024).

Within social ecological frameworks, children’s physical activity is understood to reflect the combined contribution of intrapersonal, interpersonal, environmental, and policy level factors ( Sallis *et al.*, 2000). At the interpersonal level, support from parents, peers, and teachers has been examined as a correlate of children’s physical activity. Parental support shows consistent associations with child physical activity in questionnaire-based studies, although effect sizes are often attenuated when activity is assessed via device-based measures ( Yao & Rhodes, 2015). When both parent and child activity are measured using accelerometry, associations with parental support are typically small, with correlation coefficients around 0.2 ( Sarker *et al.*, 2015; Trost & Loprinzi, 2011). Peer support has also been associated with physical activity in pre-adolescents and adolescents, although findings vary by age group, measurement approach, and activity context ( Khan *et al.*, 2020). Teacher support has been shown to increase school time physical activity in intervention settings, but its independent association with total daily activity measured by accelerometry is often weak or non-significant ( Sallis *et al.*, 2002; Sheridan *et al.*, 2014).

At the family level, parent and child physical activity are frequently associated. Studies using both self-reported and device-based measures consistently report positive correlations between parent and child activity levels ( Bringolf Isler *et al.*, 2018). These associations are often discussed in literature in terms of parental modelling, broadly defined as the co-occurrence of parental and child activity behaviors within the family context ( Bois *et al.*, 2005). However, parent–child resemblance in physical activity may arise from multiple sources, including shared routines, shared environments, and genetic relatedness. Evidence from genetically informed designs indicates that a substantial proportion of parent–child similarity in physical activity is attributable to genetic and shared environmental factors, limiting causal interpretation of behavioral pathways in observational studies ( de Geus et al., 2023).

The intensity of physical activity is associated with distinct health outcomes. Moderate intensity physical activity (MPA, 3.0–5.9 metabolic equivalents) is linked to weight management, glycemic regulation, and lipid metabolism, whereas vigorous intensity physical activity (VPA, ≥ 6.0 metabolic equivalents) provides additional benefits for cardiorespiratory fitness and musculoskeletal health ( Herrmann *et al.*, 2024; Poon *et al.*, 2021; WHO, 2020). Regular participation in MPA is consistently associated with reductions in adiposity and improvements in metabolic risk factors, while VPA and interval-based activity elicit greater gains in aerobic capacity and glucose control ( Gallardo Gómez *et al.*, 2024; Mendes *et al.*, 2019; Syeda *et al.*, 2023). High impact activities characteristic of VPA, such as running and jumping, stimulate osteogenesis and support the accrual of peak bone mass, a key determinant of fracture risk later in life ( Chevalley & Rizzoli, 2022; Miao *et al.*, 2025; Ng *et al.*, 2023).

Accurate assessment of activity intensity is therefore essential. Waist worn accelerometry provides time resolved measurement of physical activity that can be classified into intensity bands using validated cut points ( Evenson *et al.*, 2008). In contrast, self-report instruments are subject to recall error and social desirability bias and show only modest agreement with accelerometer derived estimates of activity ( Duncan *et al.*, 2005).

Most research examining associations with children’s physical activity aggregates MPA and VPA activity into a single MVPA construct, as highlighted in the meta-analysis by Petersen et al. (2020a). This practice may obscure intensity specific association patterns. Evidence from studies that separate activity by intensity indicates that parent–child resemblance is typically stronger for MPA than for VPA, while associations involving VPA are weaker and more variable (Petersen *et al.*, 2020a). These differences suggest that MPA and VPA represent distinct behavioral constructs rather than interchangeable components of a single outcome. Notably, VPA confers substantially greater health benefits per minute of activity compared with MPA, including stronger associations with reductions in all-cause mortality ( Biswas *et al.*, 2025). Despite this, intensity specific associations remain underexplored, particularly in studies that combine device-based measures of physical activity with questionnaire-based assessments of interpersonal factors.

Children aged 9 to 12 years represent a developmental stage characterized by increasing autonomy alongside continued dependence on family and school environments ( Yao & Rhodes, 2015). During this period, children’s physical activity patterns are shaped primarily within the home and school contexts, while peer related activity becomes more prominent but remains variable. Empirical evidence indicates that parent–child associations in physical activity are more consistently observed at this age than peer or teacher associations, although the strength and nature of these associations vary by activity intensity and measurement approach ( Li-juan *et al.*, 2017).

The present study draws on a subset of data from the European COST Action DE-PASS (Determinants of Physical Activities in Settings), which aims to identify and measure factors associated with physical activity behaviors across the lifespan and in different settings. One DE-PASS working group focused specifically on children aged 9 to 12 years and developed harmonized protocols for device based physical activity assessment and questionnaire-based measurement of interpersonal factors across participating countries ( Palmeira *et al.*, 2024). These procedures were designed to ensure methodological consistency and cross-national comparability. While the broader DE-PASS project addresses multiple research questions, the present study focuses on an intensity specific analysis that has not been examined in the main consortium outputs.

In this study, accelerometry was used to quantify daily MPA and VPA in children and their parents, and validated questionnaires were used to assess perceived parental, peer, and teacher support ( Palmeira *et al.*, 2024). Analyses focused on describing intensity specific associations between child and parent physical activity and between interpersonal support measures and child physical activity outcomes. The aims were to compare the strength of associations for MPA and VPA, to examine whether interpersonal support variables differed in their associations across activity intensities, and to assess whether separating MPA and VPA provided clearer insight than aggregated MVPA. In addition, sex stratified analyses were conducted to examine whether associations between parent and child MPA and VPA differed between boys and girls. Further analyses examined whether these associations differed by household socioeconomic context, as indicated by financial indicators.

## Methods

### Study design and setting

This cross-sectional study was conducted in primary schools across three European countries (Belgium, Czechia, and Ireland) between September 2023 and October 2024. Ethical approval for this study was obtained independently in each participating country. In Ireland, approval was obtained from the Research Ethics Committee of the Technological University of the Shannon (Ref. 20221201). In Belgium, approval was granted by the Ethics Committee of Ghent University (Ref. 2023-106). In Czechia, approval was provided by the Ethics Committee of the Faculty of Physical Culture, Palacký University Olomouc (Ref. No. 90/2023). All procedures were conducted in accordance with the Declaration of Helsinki, and written informed consent was obtained from parents/guardians and assent from children prior to participation. All procedures followed the DE-PASS Proof-of-Concept Standard Operating Procedures (SOP), and the reporting adhered to the Strengthening the Reporting of Observational Studies in Epidemiology (STROBE) guidelines ( von Elm *et al.*, 2007) (In supplementary 2).

### Participants

Children aged 9–12 years without diagnosed mental or physical conditions likely to alter habitual PA were eligible, provided their primary caregiver (the parent or guardian most familiar with their daily routines) also consented to participate. Recruitment was school-based (both public and private) in collaboration with school principals, initiated by a researcher-led presentation to the parent community; interested families returned signed consent forms prior to data collection.

### Data collection procedures

From each family, one study child, one primary caregiver, and one sibling (aged 8–17 years) were invited to participate, provided written informed consent (caregiver and siblings aged ≥18 years) and assent (children and younger siblings) were obtained. Families were allocated anonymized ID codes to ensure confidentiality and facilitate data linkage across measures. All procedures were approved by local ethics committees prior to implementation.

At the scheduled data collection session, families attended a face-to-face meeting with trained researchers, usually hosted in the school environment. During this visit, general family information was collected, and children’s height and weight were measured following standardized anthropometric protocols. Both the study child and primary caregiver completed validated determinant questionnaires, with assistance provided if required. To capture device-based physical activity behaviors, accelerometers were distributed to the study child, one sibling, and the primary caregiver, along with an accelerometer diary to record wear times, removals, and structured physical activity. Participants were instructed to wear the devices for eight consecutive days. Families received a study care pack containing all instructions, consent forms, and return materials. Reminder text messages were sent to caregivers on days three, four, and eight to support compliance and timely return of devices and documentation. Returned data were checked for completeness before entry into the centralized DE-PASS database.

### Accelerometer protocol

Participants wore an ActiGraph wGT3X-BT monitor (ActiGraph Corp., Pensacola, FL) affixed to a latex-free elastic belt at the right hip for eight days, removing it only for water-based activities or sleep. The ActiGraph devices were initialized using the ActiLife 6.13.4 software to sample at a 30 Hz rate. All family members received an illustrated diary and optional SMS reminder on days three and four to reinforce wear compliance. Devices were initialized with a delayed start (03:00 on the second day) and programmed for automatic stop after eight days. Unique DE-PASS ID codes were entered during initialization to ensure correct data linkage.

Upon return, raw.agd and.gt3x files were downloaded following the DE-PASS folder structure and naming convention. Data were processed with a 10-s epoch, three-axis acceleration, step count, light sensor and inclinometer channels. For children, non-wear was defined by the Troiano algorithm ( Troiano *et al.*, 2008) as ≥ 60 minutes consecutive zero counts with allowance for up to 2 minutes nonzero interruptions. Valid days required ≥ 480 minutes of wear time, with ≥ 3 valid weekdays and ≥ 1 valid weekend day required to meet the inclusion criteria. For parents, non-wear time was defined as ≥ 60 minutes consecutive zero counts. Files were examined for MPA and VPA in ActiLife using the Evenson *et al.* (2008) cut-points for children and the Troiano *et al.* (2008) cut-points for parents. Average daily MPA and VPA were calculated across valid days. This protocol was adapted from Decelis *et al.* (2014). All downloaded data were securely stored in GDPR-compliant institutional servers following DE-PASS protocols, with dual verification and password-protected master ID linkage files.

### Support assessment protocol

Both parents and children completed questionnaires. The child survey was an adapted version of the Parental Encouragement and Support for Children’s PA scale (PEACH; Ommundsen *et al.*, 2008). The parent survey comprised socio-demographic items from the Health Behaviour in School-aged Children questionnaire (HBSC; Roberts *et al.*, 2009) filled by the parent. Both questionnaires followed the adaptation procedure described by Palmeira *et al.* (2024) and were delivered either on paper. Children PEACH questionnaire yielded three subscales: parental support, peer support and teacher support. Responses were recorded on a four-point Likert scale (1 = Hardly ever or never to 4 = Every day). The original version of the instrument demonstrated good internal consistency (Cronbach’s α = .72–.89) and acceptable test–retest reliability (ICC = .65–.80) as reported in ( Palmeira *et al.*, 2024).

Missing or incomplete questionnaires were first addressed on site by the researcher’s; any forms still unfinished or unreturned prompted a follow-up phone call. Instruments that remained incomplete after these recovery steps were excluded from analysis in accordance with the SOP.

### Statistical methods

Participants with no valid child and parent accelerometry data or no corresponding child questionnaire data were removed from further analyses. Questionnaire responses for parental, peer, and teacher support were coded on an ordinal scale (1 = “Hardly ever or never” to 4 = “Every day”), and subscale scores were initially computed as the mean of their respective items (five for parental support and three each for peer and teacher support). Given the low internal consistency observed for parental and teacher support, analyses focusing on these constructs relied on individual items rather than composite scores. MPA and VPA data were transformed (square root) to normalize data and Shapiro–Wilk tests were used to assess the normality of each variable’s distribution, while Levene’s test was used to assess equality of variances, as recommended by Field (2013). The distribution of VPA remained non-normal after square root transformation. Average daily MPA and VPA (minutes per day) were calculated by dividing the total accumulated minutes by the number of valid observation days.

To identify potential socioeconomic confounders, bidirectional associations between each support variable, PA outcome (MPA and VPA minutes/day, % of total) and a set of candidate covariates were examined. Candidates were screened via point-biserial correlation using Tabachnick & Fidell, 2007 recommendations (full results in supplementary 1, socioeconomic questions in Table 1); only variables showing p < .05 associations with both an independent (support or Parent MPA and VPA) and a dependent (child MPA and VPA) variable were retained for adjustment. Child sex, country and age were directly included as covariates, given their established role as potential confounders ( Carson *et al.*, 2020; Rodríguez-Rodríguez *et al.*, 2020). Regression models were then estimated with and without these covariates to assess their impact on effect estimates.

**Table 1.** Demographic, Socioeconomic, and Accelerometry-Derived Physical Activity Characteristics of child–parent Dyads.

Characteristic	Child (N = 172)	Parent (N = 172)
**Demographics**		
**Age, mean (SD), years**	10.9 (1.6)	42.3 (5.8)
**Country of origin, n (%)**		
**Belgium**	59 (34.3)	
**Czechia**	67 (39.0)	
**Ireland**	46 (26.5)	
**Sex, n (%)** ^**#**^		
**Male**	86 (50.0)	40 (23.3)
**Female**	83 (48.3)	129 (75.0)
**Education ≥ Bachelor’s, n (%)**	—	123 (71.5)
**Socioeconomic**		
**Cars owned, n (%)**		
**0**	—	3 (1.7)
**1**	—	44 (25.6)
**2**	—	110 (64.0)
**≥ 3**	—	8 (4.7)
**Own room, n (%)**		
**Yes**	133 (77.3)	—
**No**	34 (19.8)	—
**Travels abroad, n (%)**		—
**None**	18 (10.5)	
**Once**	75 (43.6)	
**Twice**	46 (26.7)	
**> Twice**	28 (16.3)	
**Computers, n (%)**		
**0**	—	4 (2.3)
**1**	—	32 (18.6)
**2**	—	41 (23.8)
**> 2**	—	90 (52.3)
**Dishwasher, n (%)**	—	No 14 (8.1) Yes 153 (89.0)
**Bathrooms, n (%)**		
**1**	80 (46.5)	
**2**	55 (32.0)	
**> 2**	32 (18.6)	
**Physical Activity, mean (SD)**		
**MPA, minutes/day**	33.3 (10.5) *	42.87 (16.4) *
**MPA (% total PA)**	4.0 (1.3)	4.9 (1.9) *
**VPA, minutes/day**	18.8 (10.8)	4.5 (8.3)
**VPA (% total PA)**	2.2 (1.2)	0.5 (0.9)
**Social Support**		
**Peer support, mean (SD)**	2.8 (0.8)	—
**Parental support items, mean (SD)**		—
**Take you to exercise**	2.8 (1.0)	—
**Watch you take part in exercise**	3.0 (1.0)	—
**Exercise or play sports with you**	3.1 (0.9)	—
**Tell you to exercise**	2.8 (1.0)	—
**Tell you that exercise is good for your health**	2.8 (1.1)	—
**Teacher support items, mean (SD)**		—
**Talk about exercise in lessons**	3.0 (1.0)	—
**Organize or play games apart from PE**	3.2 (0.9)	—
**Tell you to exercise or play sports**	2.5 (0.9)	—

* Statistically significant difference between Male and Female. **Note:** Totals aren’t 100% since some entries were blank.

# Three participants did not answer

Descriptive statistics (means, standard deviations, and ranges for continuous variables; frequencies and percentages for categorical variables) characterized the sample and key measures. Correlations involving Support as the independent variable were calculated using Spearman’s rank-order coefficient, and those with Parent MPA and VPA as the independent variable used Pearson’s product-moment coefficient; for any variables that violated normality or homoscedasticity assumptions required by Pearson’s method, Spearman’s coefficient was applied instead.

Internal consistency of each support subscale in this study was assessed using McDonald’s omega (ω) ( McDonald, 1999), which revealed variable internal consistency. McDonald’s omega was acceptable for peer support (ω = .70) but lower for parental support (ω = .51) and teacher support (ω = .38). Since teacher and Parental support dimensions cannot be used as a single determinant, individual questions were used for the correlation analysis.

Post-hoc analyses were conducted by removing outliers from the parent–child MPA and VPA dataset, defined as values falling outside the mean ± 2 standard deviations. This approach aimed to normalize the distribution and explore whether excluding extreme cases (e.g. parents with exceptionally high VPA) would change the association patterns. All relevant variables were reanalyzed using the square root transformation procedures previously described.

Data on MVPA was not included in the present analyses, as this study was designed to focus specifically on distinguishing between MPA and VPA. Analyses of MVPA will be conducted in future work.

All analyses were conducted using JASP version 0.19.2 with a two-tailed α = .05.

## Results

### Descriptive statistics

**Figure 1.**
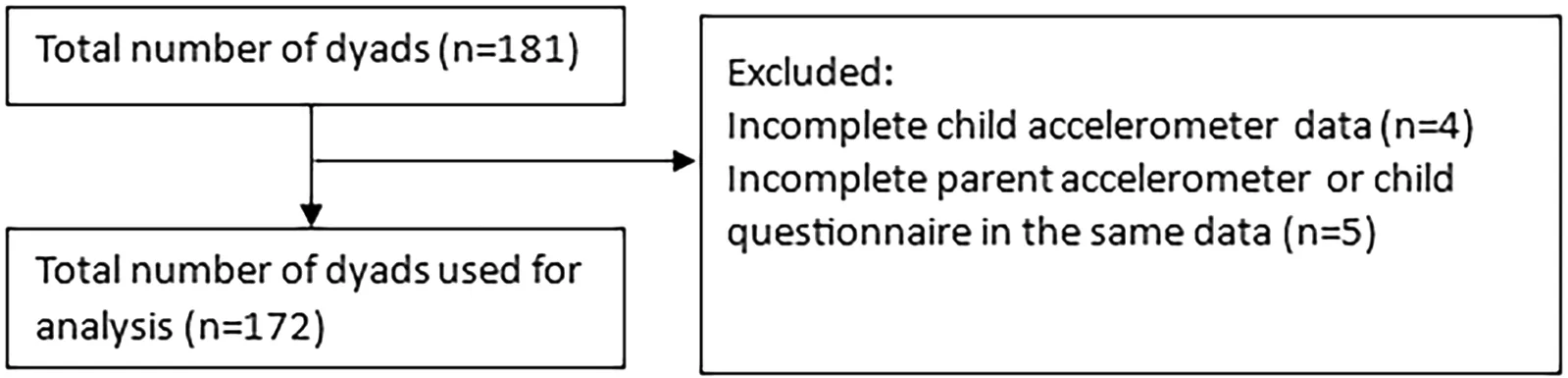
Dyads flow chart.

There were originally 181 dyads, resulting in a final sample size of 172 after removing dyads that did not meet the eligibility criteria ( Figure 1). Sample characteristics and accelerometry outcomes for the 172 child–parent dyads included in the final analysis are presented in Table 1. A post-hoc analysis was conducted using G*Power version 3.1.9.7 for a Two-Tailed Point biserial model reaching 80% for|ρ|=0.19. While children were closely matched in age as a design feature, parents likewise showed relatively low age variability. The sample showed a balanced distribution across countries, child sex was evenly represented, whereas the parental sample was predominantly female. The overall group is highly educated and of a high socioeconomic status. In terms of normality, Parent VPA in minutes and % were not normally distributed, even after transformation, which remained right skewed. In terms of sex differences, they were only observed in accelerometer determined MPA in both child and parents.

### Correlation analysis

Pearson correlation analyses between parent and child PA ( Table 2) demonstrated small to moderate positive associations across corresponding intensity levels (e.g., Parent MPA minutes were associated with children MPA, but Parent VPA was not associated with children MPA). When controlling for child sex, the correlation between child MPA minutes and parental VPA minutes became significant (ρ = .156, 95% CI [0.004, 0.295], p = .043).

**Table 2.** Pearson correlation matrix depicting associations between parent and child MPA and VPA metrics.

	Parent MPA (minutes)	Parent MPA (%)	Parent VPA (minutes) ^a^	Parent VPA (%) ^a^
**Child MPA (minutes)**	**.298** [.154, .429] < .001	**.253** [.107, .389] < .001	.146 [–.005, .290] .058	.141 [–.010, .285] .068
**Child MPA (%)**	**.291** [.147, .423] < .001	**.342** [.202, .468] < .001	.032 [–.119, .181] .682	.045 [–.106, .194] .559
**Child VPA (minutes)**	.092 [–.059, .239] .233	.030 [–.121, .180] .698	**.240** [.093, .377] .002	**.230** [.082, .367] .003
**Child VPA (%)**	.091 [–.060, .238] .239	.081 [–.071, .228] .295	**.198** [.049, .338] .010	**.197** [.048, .338] .010

^a^ Spearman correlation.

**Note:** Results are presented as correlation coefficients with corresponding 95% confidence intervals and p-values.

Adjustment for the tested confounders did not alter the statistical significance of the associations. A sex-stratified sub-analysis was conducted. Among boys, correlation patterns closely mirrored those observed in the overall sample. Among girls, correlations for MPA were consistent with those observed in the overall and boys’ analyses; however, the association between child VPA and parent VPA was not statistically significant across any of the four parent–child combinations. The full correlation matrix with partial correlations can be found in supplementary 1. Associations between child-reported social support and PA ( Table 3) were generally nonexistent to weak. After adjusting for child sex, some additional significant associations emerged. The correlation between the item ‘Exercise or play sports with you’ and child MPA % increased (ρ = 0.188, 95% CI [0.041, 0.337], p = 0.014). Furthermore, new significant associations with ‘Exercise or play sports with you’ were observed for child MPA minutes (ρ = 0.167, 95% CI [0.022, 0.301], p = 0.030), and child VPA % (ρ = 0.157, 95% CI [0.004, 0.299], p = 0.041).

**Table 3.** Spearman correlations between child-reported social support (parental, peer, teacher) and children’s MPA and VPA outcomes.

	Peer Support	(Parent) Take you to exercise	(Parent) Watch you take part in exercise	(Parent) Exercise or play sports with you	(Parent) Tell you to exercise	(Parent) Tell you that exercise is good for your health	(Teacher) Talk about exercise in lessons	(Teacher) Organize or play games apart from PE	(Teacher) Tell you to exercise or play sports
**Child MPA (minutes)**	.035 [–.127, .194] .649	.104 [–.059, .255] .173	.007 [–.166, .145] .929	.122 [–.014, .267] .110	.012 [–.135, .162] .872	.061 [–.077, .207] .424	–.068 [–.221, .088] .375	–.137 [–.273, .004] .074	–.025 [–.178, .124] .745
**Child MPA (%)**	.039 [–.117, .184] .614	.017 [–.145, .168] .824	–.109 [–.266, .050] .154	**.159** [.022, .309], .037	–.031 [–.176, .121] .686	–.013 [–.151, .131] .867	–.098 [–.246, .057] .201	–.037 [–.182, .113] .632	–.025 [–.166, .112] .748
**Child VPA (minutes)**	.011 [–.147, .163] .889	.024 [–.134, .180] .757	–.009 [–.160, .148] .907	.117 [–.033, .263] .125	–.022 [–.176, .118] .775	.011 [–.131, .167] .883	.020 [–.141, .168] .799	–.096 [–.240, .052] .210	.014 [–.148, .168] .857
**Child VPA (%)**	-.003 [–.162, .141] .969	-.035 [–.187, .124] .651	–.058 [–.207, .100] .448	.140 [–.014, .288] .067	–.034 [–.184, .112] .662	–.035 [–.170, .114] .650	.011 [–.151, .163] .886	–.044 [–.192, .111] .568	.018 [–.135, .171] .816

**Note:** Results are presented as correlation coefficients with corresponding 95% confidence intervals and p-values.

When controlling for socioeconomic determinants, specifically questions “Cars owned” and “Bathrooms”, the results were similar, with some already marginally significant values losing significance. The complete correlation matrix with partial correlations is available in supplementary 1.

Removing outlier cases did not materially change the parent–child PA correlations. However, after outlier removal, peer support showed a new, weak significant correlation with child VPA minutes (ρ = .171, 95% CI [0.006, 0.327], p = .049).

## Discussion

This study examined intensity-specific associations between parent and child physical activity and between interpersonal support and children’s physical activity, using device based measured MPA and VPA. Consistent with the primary aim, associations differed by activity intensity. Parent–child associations were stronger for MPA than for VPA, with parental MPA showing a close to moderate correlation with child MPA, while associations between parental and child VPA were weaker. This pattern is consistent with previous accelerometer-based findings reporting stronger parent–child resemblance for MPA than for VPA ( Sigmundová *et al.*, 2024) and supports the interpretation that MPA and VPA may represent distinct behavioral constructs.

The observed parent–child associations should be interpreted as evidence of familial similarity rather than as evidence of parental influence. As discussed by de Geus (2023), parent–offspring correlations in physical activity reflect a mixture of shared environmental exposure and genetic relatedness, and observational designs cannot disentangle these sources of resemblance. The present findings therefore indicate co-occurrence of parent and child activity patterns within families, but do not allow inference regarding behavioral transmission or modelling mechanisms. This interpretation is consistent with genetically informed research showing that both genetic and shared environmental factors contribute to variation in physical activity during childhood, while the relative contribution of specific pathways cannot be isolated without family-based or twin designs ( de Geus, 2023).

MPA, such as walking or casual play, is more accessible for co-participation or co-occurrence within family routines ( Gustafson & Rhodes, 2006), and adults are more likely to engage in it themselves ( Trost *et al.*, 2003; Yao & Rhodes, 2015). In contrast, parental VPA in the present study showed a non-normal distribution with a high proportion of values close to zero, while child VPA was normally distributed. This adult–child mismatch in the empirical distribution of VPA supports the interpretation that VPA and MPA behave as distinct behavioral and statistical constructs in intergenerational analyses, rather than interchangeable components of a single MVPA metric. Aggregating parental MPA and VPA into MVPA may therefore introduce noise and obscure intensity-specific associations, which may help explain attenuated or inconsistent findings in studies relying on MVPA or broader physical activity composites (Petersen *et al.*, 2020a; Petersen *et al.*, 2020b). In the present study, this distributional pattern persisted even after removing potential outliers, suggesting that combining parental VPA with normally distributed parental MPA is likely to attenuate associations and reduce statistical power, yielding less valid and less interpretable results than intensity-specific analyses.

Sex-stratified analyses indicated that associations between parental and child MPA were broadly comparable across boys and girls, whereas associations between parental and child VPA were not statistically significant among girls. These findings are consistent with previous reports of sex-specific differences in parent–child physical activity associations, particularly for higher-intensity activity ( Li-juan *et al.*, 2017). However, these results should be interpreted cautiously. The parental sample consisted predominantly of mothers, which may have limited the ability to detect associations related to paternal activity, particularly for VPA. In addition, the low prevalence of parental VPA further constrains inference regarding sex-specific patterns for VPA.

The analytical distinction between MPA and VPA has implications beyond statistical considerations. VPA appears to confer disproportionate health benefits per unit time compared with MPA, including stronger associations with reduced all-cause mortality and cardiometabolic risk ( Biswas *et al.*, 2025). Aggregating MPA and VPA into a single MVPA outcome may therefore obscure associations relevant to the most health-efficient activity intensity. The present findings support analyzing MPA and VPA separately when examining familial and interpersonal associations, particularly in studies relying on accelerometer-derived measures, not only for analytical reasons but also because MPA and VPA are associated with different health outcomes.

In line with the first aim concerning interpersonal support, perceived parental support in the form of co-participation was positively associated with children’s MPA. This finding aligns with previous work identifying shared activity as a consistent correlate of child physical activity ( Pyper *et al.*, 2016). MPA may be more feasible for co-participation due to lower physical demands, fewer safety concerns, and better alignment with adult fitness levels ( Sun *et al.*, 2013). While this association does not imply causation, it suggests that shared moderate-intensity activities are a salient feature of the family context in this age group. At the same time, prior work indicates that parental support alone may be insufficient to overcome broader environmental or motivational barriers to activity, highlighting the need for multilevel approaches ( Trost *et al.*, 2003).

In terms of examining associations between peer support and children’s VPA, it was not supported in the primary analyses. Peer support was not significantly associated with VPA in this sample of 9- to 12-year-olds. This finding is consistent with evidence suggesting that peer influences on physical activity become more prominent later in adolescence ( Beets *et al.*, 2010; Davison & Jago, 2009). A post hoc exploratory analysis conducted after removal of extreme VPA values revealed a weak association between peer support and child VPA. However, this result should be interpreted cautiously given its exploratory nature, sensitivity to data exclusion, small sample size and possibility of false positives (α= 5%). Overall, the findings suggest that peer support is not a major correlate of VPA at this developmental stage.

In the present study, teacher support was not associated with children’s PA at any intensity. Although intervention studies report increases in PA following structured teacher-led programmes (e.g., Eather *et al.*, 2013), evidence from observational research is inconsistent. The meta-analysis by Lin et al. (2024), based largely on correlational designs, found small and frequently non-significant associations between school or teacher support and PA, with stronger relationships observed for peer and family support. Similarly, the review by Wright et al. (2024) indicated that associations between educator behaviors and MVPA varied according to the type and measurement of support, with limited consistency across studies. In a synthesis of recess correlates, Zhu et al. (2025) reported that adult social or organizational factors were less consistently related to PA than environmental or individual characteristics.

These findings are compatible with the null associations observed in the current multi-country sample and suggest that, in non-intervention contexts, teacher support may not be a robust correlate of children’s PA. Taken together, the findings indicate that interpersonal associations with children’s physical activity differ by intensity and context. Parent–child resemblance is more consistently observed for MPA than for VPA, while peer and teacher support show limited associations in this age group. These results underscore the importance of distinguishing between activity intensities when examining familial and interpersonal correlates and highlight the need to distinguish association from mechanism in observational research.

## Limitations

This study has several limitations that should be considered when interpreting the findings. The cross-sectional design precludes causal inference and does not allow conclusions about the directionality of the observed associations. Parent–child resemblance in physical activity may reflect a combination of shared environments and genetic relatedness, and the present design cannot disentangle these sources of similarity or isolate specific behavioral pathways, such as parental modelling.

The sample was drawn primarily from European countries and consisted largely of families from higher socioeconomic backgrounds, with mothers comprising the majority of participating parents. This sample composition may limit the generalizability of the findings to families from different cultural, socioeconomic, or family structures, and may also have reduced variability in socioeconomic indicators, limiting the ability to detect differences or stratified patterns related to socioeconomic context. In addition, the predominance of mothers in the parental sample may have constrained the ability to detect sex-specific parent–child associations, particularly those related to paternal activity patterns.

Although the study focused on interpersonal factors, other contextual influences, such as the built environment, neighborhood characteristics, and school-level policies were not included. In addition, children were recruited from a limited number of schools, in some cases from the same classes, raising the possibility of clustering effects, particularly for teacher support variables. Because school-level identifiers were not available due to data protection constraints, it was not possible to formally account for clustering in the analysis.

While the parental, peer, and teacher support instruments have demonstrated acceptable reliability in previous research, the internal consistency of the parental and teacher support subscales was suboptimal in the present sample. Consequently, analyses relied on individual questionnaire items rather than composite scores, which may have limited the ability to capture the full breadth of these interpersonal constructs. Several factors may have affected measurement accuracy, including the age of the participants, the sample size, and the risk of social desirability in child self-report. These issues should be considered when interpreting the findings. Although procedures described in the Methods were implemented to minimize bias, they cannot fully eliminate it.

Finally, although the sample size was sufficient to detect moderate associations, the study was underpowered to identify very weak associations. Some secondary analyses, including stratification by sex and socioeconomic indicators, were exploratory in nature and should therefore be interpreted cautiously. In addition, the restricted age range limits the applicability of the findings to other developmental stages, particularly adolescence, where peer and school influences on physical activity may operate differently.

## Conclusions

This study underscores the importance of distinguishing between physical activity intensities when examining interpersonal associations with children’s activity.Associations between parent and child physical activity differed by intensity, with more consistent parent–child resemblance observed for MPA than for VPA. In contrast, peer and teacher support showed limited or no associations with children’s physical activity in this age group.

The findings further demonstrate that aggregating MPA and VPA into a single MVPA construct may obscure intensity-specific association patterns and reduce interpretability in intergenerational analyses. Treating MPA and VPA as distinct outcomes potentially provides clearer insight into how familial and interpersonal factors co-occur with children’s activity behaviors.

Future research should prioritize longitudinal designs and refined, validated measurement instruments that distinguish clearly between MPA and VPA, to better characterize how intensity-specific associations evolve across developmental stages. From an applied perspective, the findings suggest that family-based approaches focusing on MPA may be particularly relevant during late childhood, given the more consistent parent–child associations observed for this intensity. Interventions that promote shared MPA within daily family routines may therefore represent a feasible and contextually appropriate strategy for supporting physical activity in this age group, while recognizing that different approaches may be required to address VPA.

### Extended data

Open Science Framework. DE-PASS MPA and VPA cross-sectional supplementary data. OSF, https://osf.io/qc8g2/?view_only=2ec73bad89674857859d142f4e6f7d7c ( Ferreira *et al.*, 2025)

This project contains the following extended data:

• Supplementary 1 (Correlation matrices: partial correlations with socioeconomic covariates).
• Supplementary 2 (STROBE checklist).

Data are available under the terms of the Creative Commons Attribution 4.0 International license (CC BY 4.0).
