# Supplementary material for: Moderate and vigorous physical activity show intensity specific familial and interpersonal association patterns in 9–12-year-olds: A cross-sectional DE-PASS study
Source: Open Res Eur. 2026 Sep 2;5:331. Originally published 2025 Oct 28. [Version 4] doi: 10.12688/openreseurope.21538.4 (PMC13586601; doi:10.12688/openreseurope.21538.4)
Supplement: Peer review history

# Reviewer report: Reviewer response for version 4

**DOI:** 10.21956/openreseurope.26828.r80189

**Published:** 2026-09-17

**Authors:** Darko Jekauc

**Affiliations:** 1 KarlsruheInstitute of Technology, Karlsruhe, Germany

I do not have any further comments.

Is the study design appropriate and does the work have academic merit?

Partly

Is the work clearly and accurately presented and does it cite the current literature?

Partly

If applicable, is the statistical analysis and its interpretation appropriate?

Partly

Are all the source data underlying the results available to ensure full reproducibility?

Partly

Are the conclusions drawn adequately supported by the results?

Partly

Are sufficient details of methods and analysis provided to allow replication by others?

Partly

Reviewer Expertise:

I have expertise in health psychology, sport and exercise psychology, and physical activity behavior research. My work focuses on psychological determinants of physical activity, health behavior change, intention-behavior relations, and the use of observational and intensive longitudinal research designs in physical activity contexts. I also have expertise in the conceptual interpretation of behavioral associations, particularly in distinguishing correlational findings from causal mechanisms.In addition, I am familiar with questionnaire-based assessment of psychosocial determinants, accelerometer-based physical activity research at the level needed to evaluate the conceptual and behavioral interpretation of the measures, and the statistical evaluation of observational studies, including correlational analyses, regression-based approaches, moderation, confounding, and reproducibility issues.Based on this expertise, I feel able to assess the theoretical framing, relevance of the research question, interpretation of parent-child and interpersonal associations, appropriateness of the observational design, adequacy of the behavioral and psychosocial measures, and the general appropriateness of the statistical analyses and conclusions. I would not claim specialist expertise in accelerometer calibration or raw accelerometry data processing, but I am able to evaluate whether the measurement protocol and interpretation are appropriate from the perspective of physical activity and health behavior research.

I confirm that I have read this submission and believe that I have an appropriate level of expertise to confirm that it is of an acceptable scientific standard.

# Reviewer report: Reviewer response for version 4

**DOI:** 10.21956/openreseurope.26828.r80190

**Published:** 2026-09-08

**Authors:** Aysegul Simsek

**Affiliations:** 1 Marmara University, İstanbul, Turkey

The authors have implemented my suggestions.

Is the study design appropriate and does the work have academic merit?

Yes

Is the work clearly and accurately presented and does it cite the current literature?

Yes

If applicable, is the statistical analysis and its interpretation appropriate?

Yes

Are all the source data underlying the results available to ensure full reproducibility?

Yes

Are the conclusions drawn adequately supported by the results?

Yes

Are sufficient details of methods and analysis provided to allow replication by others?

Yes

Reviewer Expertise:

Pediatric Nursing

I confirm that I have read this submission and believe that I have an appropriate level of expertise to confirm that it is of an acceptable scientific standard.

# Reviewer report: Reviewer response for version 3

**DOI:** 10.21956/openreseurope.26500.r77122

**Published:** 2026-07-10

**Authors:** Darko Jekauc

**Affiliations:** 1 KarlsruheInstitute of Technology, Karlsruhe, Germany

The revised manuscript has improved substantially. In particular, I appreciate the clearer distinction between primary, secondary, and exploratory analyses, the addition of a formal comparison between parent child correlations for MPA and VPA, the use of Benjamini Hochberg correction for the support item analyses, the revised covariate strategy, the clarification that the primary analyses were correlational rather than regression based, and the more cautious framing of parent child associations as familial resemblance rather than causal parental influence.

 Overall, the manuscript is now considerably more precise and scientifically stronger. However, I still have several reservations that should be addressed before I can consider the article fully scientifically sound.

 First, the title and some parts of the abstract still seem stronger than the empirical evidence supports. The revised analyses show that parent child resemblance was numerically larger for MPA than for VPA, but the formal comparison between intensities was not statistically significant. Therefore, the statement that moderate and vigorous physical activity “show distinct familial and interpersonal association patterns” remains somewhat too strong. A more cautious title would better reflect the results, for example by referring to “intensity specific correlates” or “intensity specific familial and interpersonal associations” rather than claiming distinct patterns.

 Second, there appears to be an inconsistency in the reporting of the formal comparison of the dependent correlations. In the manuscript, the comparison for percentage of total activity is reported as Δρ = .16, 95% CI [.01, .34], p = .06. A 95 percent confidence interval that does not include zero is difficult to reconcile with a two tailed p value of .06. In the author response, the interval is reported as 95% CI [minus .01, .34], which would be consistent with the nonsignificant p value. This should be checked and corrected, because it concerns one of the central statistical results of the paper.

 Third, the Plain Language Summary should be revised to align with the cautious interpretation in the main manuscript. Several statements still imply causal influence or intervention effectiveness, for example that parents are the strongest role models, that the best way to help children be more active is through family involvement, or that children were more active when their parents were active. The main manuscript now appropriately emphasizes that the study is cross sectional and cannot distinguish genetic, shared environmental, or behavioral transmission mechanisms. The Plain Language Summary should use the same level of caution and refer to associations or familial resemblance rather than influence or causal role modelling.

 Fourth, the applied implications should remain cautious throughout the manuscript. The finding that parental co participation showed only a weak association with child MPA and did not survive correction for multiple testing should not be used as a strong basis for recommending family-based approaches. It would be more appropriate to state that family-based approaches focusing on MPA may be a plausible direction for future longitudinal or intervention research, rather than an implication directly supported by the present findings.

 Finally, the data availability statement is improved, but full reproducibility remains only partly ensured. I understand that individual level DE PASS data are governed by the consortium and may not be directly shareable by the authors. However, aggregated descriptive statistics and correlation matrices do not allow full independent reproduction of the analyses. This does not necessarily preclude acceptance, but the limitation should be stated explicitly and transparently.

Is the study design appropriate and does the work have academic merit?

Partly

Is the work clearly and accurately presented and does it cite the current literature?

Partly

If applicable, is the statistical analysis and its interpretation appropriate?

Partly

Are all the source data underlying the results available to ensure full reproducibility?

Partly

Are the conclusions drawn adequately supported by the results?

Partly

Are sufficient details of methods and analysis provided to allow replication by others?

Partly

Reviewer Expertise:

I have expertise in health psychology, sport and exercise psychology, and physical activity behavior research. My work focuses on psychological determinants of physical activity, health behavior change, intention-behavior relations, and the use of observational and intensive longitudinal research designs in physical activity contexts. I also have expertise in the conceptual interpretation of behavioral associations, particularly in distinguishing correlational findings from causal mechanisms.In addition, I am familiar with questionnaire-based assessment of psychosocial determinants, accelerometer-based physical activity research at the level needed to evaluate the conceptual and behavioral interpretation of the measures, and the statistical evaluation of observational studies, including correlational analyses, regression-based approaches, moderation, confounding, and reproducibility issues.Based on this expertise, I feel able to assess the theoretical framing, relevance of the research question, interpretation of parent-child and interpersonal associations, appropriateness of the observational design, adequacy of the behavioral and psychosocial measures, and the general appropriateness of the statistical analyses and conclusions. I would not claim specialist expertise in accelerometer calibration or raw accelerometry data processing, but I am able to evaluate whether the measurement protocol and interpretation are appropriate from the perspective of physical activity and health behavior research.

I confirm that I have read this submission and believe that I have an appropriate level of expertise to confirm that it is of an acceptable scientific standard, however I have significant reservations, as outlined above.

# Response

**Published:** 2026-07-30

**Authors:** Flávio Ferreira

**Affiliations:** Lusófona University of Humanities and Technologies Research Center on Sports Physical Education Exercise and Health, Lisbon, Lisbon, Portugal

*Manuscript: “Moderate and vigorous physical activity show intensity-specific familial and interpersonal associations in 9–12-year-olds: A cross-sectional DE-PASS study” (Open Research Europe, 5-331).* We thank Dr. Jekauc for the continued careful and constructive assessment, and for recognizing the improvements made in the previous revision. We have addressed each of the five remaining points below. 

**1. Title and abstract stronger than the evidence supports.** ***Reviewer’s comment.*** *The formal comparison between intensities was not statistically significant, so “show distinct familial and interpersonal association patterns” is too strong; a more cautious wording such as “intensity-specific” would better reflect the results.*

**Response. **We agree. The title now reads “Moderate and vigorous physical activity show intensity-specific familial and interpersonal associations in 9–12-year-olds,” adopting the reviewer’s suggested framing. In the abstract we replaced “differed by activity intensity” with “varied by activity intensity,” “support treating MPA and VPA as distinct outcomes” with “support examining MPA and VPA as intensity-specific outcomes,” and “show that … differ by intensity” with “indicate that … vary by intensity.” Corresponding statements in the Introduction, Discussion, and Conclusion were softened from “distinct constructs” to “intensity-specific constructs” and from “supports” to “is consistent with.”

**2. Inconsistency in the reported confidence interval.** ***Reviewer’s comment.*** *The manuscript reports Δρ = .16, 95% CI [.01, .34], p = .06, but a CI that excludes zero is difficult to reconcile with a two-tailed p = .06; the author response reported [−.01, .34].*

**Response. **The correct value is Δρ = .16, 95% CI [−.01, .34], p = .06. The lower bound is negative, so the interval includes zero, consistent with the non-significant two-tailed p value.

**3. Plain Language Summary should match the cautious interpretation.** ***Reviewer’s comment.*** *Several statements still imply causal influence or intervention effectiveness (for example, that parents are the strongest role models, that the best way to help children be more active is through family involvement, or that children were more active when their parents were active).*

**Response. **We have revised the Plain Language Summary to use the same association-based, cautious language as the main text. We removed statements implying that parental activity makes children more active (for example, “children tended to be more active when their parents were active” and “if parents often walked or cycled, children were more likely to do the same”) and replaced them with a description of familial resemblance, together with an explicit statement that the study is cross-sectional and cannot show what causes what or separate genetic, shared-environmental, and behavioral pathways. We also removed the suggestion that family involvement is “a promising way to support children’s activity,” reframing it as a possible direction for future research. We made most of the plain summary to match the actual current conclusion of the manuscript.

**4. Applied implications should remain cautious throughout.** ***Reviewer’s comment.*** *Parental co-participation showed only a weak association with child MPA and did not survive correction for multiple testing, so it should not be a strong basis for recommending family-based approaches; these should be framed as a plausible direction for future research.* **Response. **We have made clearer in discussion child and parent VPA dataset differences which has direct implication in statistical analysis warrinting caution “Because parental VPA was low and with a high frequency of values at or near zero, its restricted variance and the resulting tied ranks reduce the reliability of the parent–child VPA correlation and the statistical power of the formal intensity comparison; the non‑significant difference should be interpreted with this limitation in mind rather than as evidence of equivalent resemblance.” In conclusion recommendations were softened and adjusted to results “From an applied perspective, these findings suggest that family-based approaches focusing on MPA of daily family routines may be a plausible direction for future longitudinal or invertention research since parental VPA is distributed differently from children’s, but more targeted studies are needed to fully corroborate this .”

**5. Data availability and reproducibility.** ***Reviewer’s comment.*** *Aggregated descriptive statistics and correlation matrices do not allow full independent reproduction of the analyses; this limitation should be stated explicitly and transparently.*

**Response. **We have added following limitation: “Datasets belong to the DE-PASS consortium and raw data can’t be shared without reasonable request to the consortium.” Also added in dataset availability that dataset can be shared when asked to the DE-PASS consortium upon reasonable request. _____ We believe these revisions address Dr. Jekauc remaining reservations while keeping the manuscript’s claims closely aligned with the data. We are grateful for the reviewer’s thorough and constructive input.

# Reviewer report: Reviewer response for version 2

**DOI:** 10.21956/openreseurope.24547.r72531

**Published:** 2026-05-23

**Authors:** Darko Jekauc

**Affiliations:** 1 KarlsruheInstitute of Technology, Karlsruhe, Germany

The manuscript addresses a relevant and timely question: whether moderate physical activity and vigorous physical activity show different familial and interpersonal association patterns in children aged 9 to 12 years. The study uses cross-sectional data from the DE-PASS project, including 172 child-parent dyads from Belgium, Czechia, and Ireland. Physical activity in both children and parents was assessed with waist-worn accelerometers, and children reported perceived support from parents, peers, and teachers. The main finding is that parent-child associations appear more consistent for moderate physical activity than for vigorous physical activity, whereas peer and teacher support show weak or no associations with children’s activity. The authors conclude that moderate and vigorous physical activity should be treated as distinct outcomes rather than aggregated into MVPA.

 Overall, the manuscript has scientific merit. The use of device-based measures in both children and parents is a clear strength, and the conceptual reframing away from causal “parental modeling" toward familial resemblance is appropriate. However, several aspects require revision before the article can be considered scientifically sound. The main concerns are that the central claim about MVPA aggregation is not directly tested, that differences between MPA and VPA associations are interpreted without formal statistical comparison, that many exploratory correlations are tested without sufficient control of multiplicity, and that reproducibility is only partly ensured.

**Is the work clearly and accurately presented, and does it cite the current literature?**

 Partly.

 The manuscript is generally well written, and the rationale for distinguishing MPA and VPA is clearly presented. The literature is current and relevant, including recent work on accelerometer-based physical activity, parent-child resemblance, social support, and intensity-specific health effects. The revised framing is also appropriate in recognizing that observational parent-child associations cannot be interpreted as evidence of causal parental modeling.

 However, the presentation should be made more precise in several respects. First, the manuscript should consistently avoid causal language such as “influence,” "modeling," or “strategies to address VPA” unless explicitly framed as speculative. The current data support associations and resemblance, not behavioral transmission or intervention effects. Second, the claim that aggregating MPA and VPA into MVPA would obscure intensity-specific patterns is too strong in the absence of an empirical MVPA analysis. This statement should either be supported by additional MVPA analyses or reformulated as a plausible methodological concern rather than a demonstrated result. Third, the distinction between familial resemblance, interpersonal support, shared environment, and behavioral influence should be maintained more consistently throughout the manuscript.

**Is the study design appropriate, and does the work have academic merit?**

 Yes, with important limitations.

 The cross-sectional design is appropriate for a descriptive and correlational study of intensity-specific associations. The use of harmonized procedures across three countries and accelerometer-based measures in both parents and children provides academic value. The manuscript makes a useful contribution by emphasizing that MPA and VPA may not be interchangeable behavioral constructs.

 Nevertheless, the academic contribution remains primarily descriptive. The study cannot explain why parent-child associations differ by intensity, nor can it disentangle shared environmental, genetic, or behavioral mechanisms. The authors acknowledge this limitation, which is important. The conclusions should remain aligned with this design and should not imply causal influence, developmental processes, or intervention efficacy.

**Are sufficient details of methods and analysis provided to allow replication by others?**

 Partly.

 The manuscript provides useful details on recruitment, eligibility criteria, accelerometer placement, wear-time criteria, epoch length, cut-points, questionnaire measures, transformation procedures, software, and general analytic strategy. These details support conceptual replication.

 However, full analytic replication would require additional information. The authors should report the exact sample size used in each correlation, because missing questionnaire or accelerometer data may vary across analyses. They should specify how missing questionnaire items were handled within each subscale or item-level analysis. The description of covariate selection and adjustment should be clarified, including which variables were included in each partial correlation or regression. The manuscript refers to regression models with and without covariates, but the reported results are mainly correlations and partial correlations. This inconsistency should be resolved. Either the regression models should be reported in full, or the methods should be rewritten to reflect that the primary analyses were correlational.

**If applicable, is the statistical analysis and its interpretation appropriate?**

 Partly.

 The use of Pearson and Spearman correlations is acceptable for an exploratory cross-sectional analysis. The use of confidence intervals is helpful, and the cautious interpretation of parent-child associations as familial resemblance rather than causal modeling is appropriate.

 Several statistical issues need to be addressed. The most important concern is that the manuscript interprets MPA associations as stronger and more consistent than VPA associations but does not formally test whether these correlations differ. Since this comparison is central to the manuscript, the authors should statistically compare the relevant dependent correlations or use an alternative model that directly tests intensity as a moderator. For example, a long-format regression model with physical activity intensity as a within-dyad factor could test whether associations differ between MPA and VPA.

 Second, the manuscript conducts many correlations across parent activity variables, child activity variables, support subscales, support items, sex-stratified analyses, socioeconomic sensitivity analyses, and outlier-excluded analyses. This creates a substantial risk of false-positive findings. The authors should clearly distinguish primary from exploratory analyses and consider correction for multiple testing or, at minimum, interpret marginal p-values very cautiously. Findings such as the weak associations emerging only after adjustment or outlier removal should not be presented as robust evidence.

 Third, the central claim that MVPA aggregation obscures intensity-specific associations is not directly tested. The authors explicitly state that MVPA was not included in the present analyses. This is problematic because the title, abstract, discussion, and conclusion make a strong argument about the limitations of MVPA aggregation. The authors should add an MVPA sensitivity analysis comparing parent-child and support associations for MVPA with those for MPA and VPA separately. If they choose not to include MVPA, they should substantially weaken the claim and state that their results suggest, rather than demonstrate, that aggregation could obscure patterns.

 Fourth, the covariate strategy is not optimal. Selecting socioeconomic covariates based on significant bivariate associations with both predictors and outcomes is data-driven and may produce unstable adjustment sets. A theory-driven covariate strategy would be preferable. At minimum, child age, child sex, country, parent sex, and a prespecified socioeconomic indicator should be considered. If the sample size limits the number of covariates, this should be stated clearly, and sensitivity analyses should be interpreted accordingly.

 Fifth, the school-based recruitment creates potential clustering, especially for teacher support variables. The authors acknowledge that school-level identifiers were not available. This limitation should be treated as more than a minor caveat, because non-independence can affect standard errors and p-values. If country or school-level information is available, the authors should consider sensitivity analyses using country fixed effects or cluster-robust approaches. If school identifiers are unavailable, the potential impact on inference should be discussed more explicitly.

 Finally, the post-hoc power analysis is of limited interpretive value. It would be more informative to focus on effect sizes, confidence intervals, and the precision of estimates. The authors should consider removing the post-hoc power statement or replacing it with a clearer discussion of detectable effect sizes and uncertainty.

**Are all the source data underlying the results available to ensure full reproducibility?**

 Partly.

 The manuscript provides an OSF link to supplementary materials, including partial correlation matrices and a STROBE checklist. This improves transparency. However, full reproducibility requires more than supplementary correlation tables. The authors should provide an anonymized analysis dataset, a codebook, and the complete analysis syntax from JASP or, preferably, R. If the full dataset cannot be shared because of ethical or data protection constraints, the manuscript should explicitly state this and describe the conditions under which data can be accessed. At present, the available materials appear insufficient to fully reproduce the reported analyses.

**Are the conclusions drawn adequately supported by the results?**

 Partly.

 The conclusion that parent-child physical activity associations differ by intensity is broadly supported by the pattern of reported correlations, although it should be strengthened by formal tests of differences between MPA and VPA associations. The conclusion that peer and teacher support show limited associations with activity in this age group is also generally supported.

 However, some conclusions are stronger than the data allow. The statement that MVPA aggregation obscures intensity-specific patterns is not directly supported unless MVPA is analyzed. The applied conclusion that family-based approaches focusing on MPA may be particularly relevant should be phrased more cautiously. The data show that parent-child MPA and parental co-participation are associated with child MPA, but they do not show that family-based interventions would increase MPA. This should be presented as a hypothesis for future intervention studies rather than a direct implication.

**Points that must be addressed to make the article scientifically sound**

 The authors should either add MVPA analyses or substantially weaken all claims that MVPA aggregation obscures relevant patterns. Given that this claim is central to the manuscript, adding MVPA as a sensitivity analysis would be the preferred solution.

 The authors should formally test whether parent-child associations differ between MPA and VPA. Numerical differences between correlations are not sufficient to support the central inference.

 The authors should clarify the analytic hierarchy by identifying primary, secondary, and exploratory analyses. They should address multiple testing, especially for the numerous support-item correlations and post-hoc subgroup analyses.

 The authors should revise the covariate strategy. A prespecified, theory-driven adjustment set would be preferable to a data-driven selection procedure based on bivariate significance.

 The authors should clarify whether regressions were conducted. If regression models were used, they should be reported. If not, references to regression models should be removed or rewritten.

 The authors should improve reproducibility by providing an anonymized analysis dataset, codebook, and analysis syntax, or by explaining why this is not possible and how the data can be accessed.

 The authors should revise the discussion and conclusion to avoid causal or intervention-oriented overstatement. Terms such as “influence,” “modelling,” and “family-based strategies” should be used only with explicit caution.

**Recommendation**

 The manuscript is promising and addresses a relevant question, but it requires revision before it can be considered fully scientifically sound. My recommendation would be approval only after revision. The required revisions are feasible and mainly concern analytic precision, reproducibility, and alignment between results and conclusions.

Is the study design appropriate and does the work have academic merit?

Partly

Is the work clearly and accurately presented and does it cite the current literature?

Partly

If applicable, is the statistical analysis and its interpretation appropriate?

Partly

Are all the source data underlying the results available to ensure full reproducibility?

Partly

Are the conclusions drawn adequately supported by the results?

Partly

Are sufficient details of methods and analysis provided to allow replication by others?

Partly

Reviewer Expertise:

I have expertise in health psychology, sport and exercise psychology, and physical activity behavior research. My work focuses on psychological determinants of physical activity, health behavior change, intention-behavior relations, and the use of observational and intensive longitudinal research designs in physical activity contexts. I also have expertise in the conceptual interpretation of behavioral associations, particularly in distinguishing correlational findings from causal mechanisms.In addition, I am familiar with questionnaire-based assessment of psychosocial determinants, accelerometer-based physical activity research at the level needed to evaluate the conceptual and behavioral interpretation of the measures, and the statistical evaluation of observational studies, including correlational analyses, regression-based approaches, moderation, confounding, and reproducibility issues.Based on this expertise, I feel able to assess the theoretical framing, relevance of the research question, interpretation of parent-child and interpersonal associations, appropriateness of the observational design, adequacy of the behavioral and psychosocial measures, and the general appropriateness of the statistical analyses and conclusions. I would not claim specialist expertise in accelerometer calibration or raw accelerometry data processing, but I am able to evaluate whether the measurement protocol and interpretation are appropriate from the perspective of physical activity and health behavior research.

I confirm that I have read this submission and believe that I have an appropriate level of expertise to confirm that it is of an acceptable scientific standard, however I have significant reservations, as outlined above.

# Response

**Published:** 2026-06-29

**Authors:** Flávio Ferreira

**Affiliations:** Lusófona University of Humanities and Technologies Research Center on Sports Physical Education Exercise and Health, Lisbon, Lisbon, Portugal

**Response to Reviewer 2** Manuscript: Moderate and vigorous physical activity show distinct familial and interpersonal association patterns in 9 to 12 year olds: A cross-sectional DE-PASS study. We thank Reviewer 2 for the thorough and constructive evaluation. We have revised the manuscript to address each point, and all changes are shown as tracked changes in the revised version.

**Points that must be addressed** **1. The MVPA aggregation claim** ***Reviewer 2:*** *The claim that aggregating MPA and VPA into MVPA would obscure intensity-specific patterns is too strong in the absence of an empirical MVPA analysis. This statement should either be supported by additional MVPA analyses or reformulated as a plausible methodological concern rather than a demonstrated result.*

**Response: **We have reformulated these statements as a plausible methodological concern rather than a demonstrated result. In the Abstract, "would have obscured" is now "may obscure"; in the Discussion, "is likely to attenuate ... yielding less valid and less interpretable results" is now "could attenuate ... and may yield less interpretable results." MVPA outcomes from the DE-PASS dataset are reserved for separate, pre-planned working-group analyses and therefore could not be reported here. The argument for analysing the intensities separately now rests on their different health implications and on the markedly different empirical distributions of MPA (normal) and VPA (non-normal, with many values near zero) observed in our data. We note that the formal comparison of parent-child resemblance across intensities (Point 2) did not reach significance; we therefore do not claim that resemblance differs by intensity, and the case for analysing the intensities separately is made on distributional and health-outcome grounds rather than on a difference in the strength of resemblance.

**2. Formal test of whether MPA and VPA associations differ** ***Reviewer 2:*** *The manuscript interprets MPA associations as stronger and more consistent than VPA associations but does not formally test whether these correlations differ. The authors should statistically compare the relevant dependent correlations.*

**Response: **We have added a formal comparison of the two dependent, non-overlapping correlations (parent-child MPA versus parent-child VPA) using Steiger's test and Zou's confidence interval, corroborated by a non-parametric bootstrap. Because parental VPA was non-normal, Spearman coefficients were used for both intensities. The difference between intensities was not statistically significant (percentage of total activity: difference in rho = .16, 95% CI [-.01, .34], p = .06; minutes per day: difference in rho = .08, p = .39). We have accordingly softened the central claim: the manuscript now states that parent-child resemblance was significant at both intensities and numerically larger for moderate activity, but that the two intensities did not differ significantly.

**3. Analytic hierarchy and multiple testing** ***Reviewer 2:*** *The authors should clearly distinguish primary from exploratory analyses and consider correction for multiple testing, especially for the numerous support-item correlations and post-hoc subgroup analyses. Findings emerging only after adjustment or outlier removal should not be presented as robust evidence.*

**Response: **We now state an explicit analytic hierarchy: primary (parent-child associations), secondary (sex-stratified and socioeconomic sensitivity analyses), and exploratory (associations of individual support items and outlier-excluded analyses). We apply the Benjamini-Hochberg false discovery rate correction to the family of support-item correlations. After correction, no support to activity association remained significant (smallest adjusted p = .85). The co-participation finding has been removed from the headline narrative and is now presented, together with the post-hoc peer-support finding, as exploratory.

**4. Covariate strategy** ***Reviewer 2:*** *Selecting socioeconomic covariates based on significant bivariate associations with both predictors and outcomes is data-driven and may produce unstable adjustment sets. A theory-driven covariate strategy would be preferable.*

**Response: **We have replaced the data-driven covariate selection with a prespecified, theory-based adjustment set: child age, child sex, country, parent sex, and parental education. Parental education was chosen as a widely used indicator of family socioeconomic status in children's physical activity research (Ziegeldorf et al., 2024). Re-estimating the associations with this prespecified set left the conclusions unchanged (parent-child MPA partial rho about .31 to .33, p < .001; parent-child VPA partial rho about .17 to .19, p < .05). The earlier screening is retained as a complementary sensitivity analysis.

**5. Regression versus correlation** ***Reviewer 2:*** *The manuscript refers to regression models with and without covariates, but the reported results are mainly correlations and partial correlations. This inconsistency should be resolved.*

**Response: **The primary analyses were correlational. We have revised the Methods so that the adjustment is described as partial correlations re-estimated with and without the covariates, consistent with the reported results, and the reference to regression models has been removed.

**6. Reproducibility** ***Reviewer 2:*** *The authors should provide an anonymized analysis dataset, a codebook, and the complete analysis syntax. If the full dataset cannot be shared because of ethical or data protection constraints, the manuscript should explicitly state this and describe the conditions under which data can be accessed.*

**Response: **The individual-level DE-PASS data are governed by the DE-PASS consortium and are not held by the authors; access may be requested from the DE-PASS committee. We have added a data-availability statement to this effect. We additionally provide, in the Open Science Framework repository, the aggregate values underlying the analyses (descriptive statistics and correlation matrices, with MVPA outcomes excluded).

**7. Causal and intervention language** ***Reviewer 2:*** *The authors should revise the discussion and conclusion to avoid causal or intervention-oriented overstatement. Terms such as influence, modelling, and family-based strategies should be used only with explicit caution.*

**Response: **The revised manuscript reframes parent-child associations as familial resemblance rather than parental influence, and explicitly states that observational data cannot disentangle genetic and shared-environmental contributions to that resemblance. Applied implications are stated cautiously, using hedged language and emphasising the need for longitudinal and intervention research rather than asserting demonstrated effects, and the comparative and aggregation claims have been softened as described under Points 1 and 2.

**Additional specific points** **Response: **Exact sample size per analysis. We now report that, owing to item-level and accelerometer missingness (pairwise deletion), unadjusted correlations were based on 169 to 172 dyads and covariate-adjusted partial correlations on 163 to 165 dyads.

**Response: **Handling of missing questionnaire items. Item-level missingness was minimal (no item had more than two missing responses); where a contributing item was missing, the subscale score was computed from the remaining items, and all analyses used pairwise deletion.

**Response: **School-level clustering. We note that country was included as a covariate, which partially accounts for between-country variation, while residual clustering at the school or class level could not be modelled because identifiers were unavailable; associations involving teacher support in particular are interpreted with this in mind.

**Response: **Post-hoc power analysis. The post-hoc power statement has been reframed as a sensitivity power analysis describing the smallest effect detectable at the present sample size, and all estimates are reported with 95% confidence intervals.

**We thank the reviewer again for these constructive comments, which we believe have substantially strengthened the manuscript, which had multiple alterations and by so had segments where wording was stale and needed improvement to meet final methods and results.**

# Reviewer report: Reviewer response for version 1

**DOI:** 10.21956/openreseurope.23298.r64482

**Published:** 2026-01-05

**Authors:** Aysegul Simsek

**Affiliations:** 1 Marmara University, İstanbul, Turkey

The article provides evidence of shared data regarding the daily life activities of parent-child pairs. Although it is a small part of a larger project, the data obtained alone demonstrates scientific validity. The language is well written. The methodology is presented in detail and is understandable. The findings are appropriately discussed in the discussion section with support from the literature. Acceptable.

Is the study design appropriate and does the work have academic merit?

Yes

Is the work clearly and accurately presented and does it cite the current literature?

Yes

If applicable, is the statistical analysis and its interpretation appropriate?

Yes

Are all the source data underlying the results available to ensure full reproducibility?

Yes

Are the conclusions drawn adequately supported by the results?

Yes

Are sufficient details of methods and analysis provided to allow replication by others?

Yes

Reviewer Expertise:

Child Health and Diseases Nursing

I confirm that I have read this submission and believe that I have an appropriate level of expertise to confirm that it is of an acceptable scientific standard.
